# QTLs for heat-induced stomatal anatomy underpin gas exchange variation in field-grown wheat

**DOI:** 10.3389/fpls.2026.1769384

**Published:** 2026-05-18

**Authors:** Edward Chaplin, Emi Tanaka, Andrew Merchant, Beata Sznajder, Richard Trethowan, William Salter

**Affiliations:** 1 School of Life and Environmental Sciences, Sydney Institute of Agriculture, The University of Sydney, Sydney, NSW, Australia; 2 Research School of Finance, Actuarial Studies and Statistics, Australian National University, Canberra, ACT, Australia; 3 School of Agriculture, Food and Wine, Waite Research Institute, University of Adelaide, Adelaide, SA, Australia; 4 The Australian Plant Phenomics Network, The University of Sydney, Narrabri, NSW, Australia

**Keywords:** field-based phenotyping, genotypic variation, heat stress, plant breeding, quantitative trait loci, stomatal anatomy, stomatal conductance, wheat

## Abstract

Stomata are central to leaf gas exchange, governing carbon uptake, water loss, and, ultimately, crop performance. However, how stomatal anatomy and physiology integrate to determine wheat heat tolerance under field conditions remains poorly understood. Here, we examined the contribution of stomatal anatomical and physiological traits in shaping wheat responses to heat stress. Across 2 years of multi-environment field trials encompassing 200 genotypes in season 1 and 50 genotypes in season 2, we examined stomatal conductance (*g_s_*), anatomical traits including stomatal size and density, and stomatal conductance operating efficiency (*g_se_*) across leaf surfaces, along with grain yield. Timely and delayed sowing treatments were used to expose anthesis to contrasting temperature regimes. Early sowing supported higher *g_s_* and *g_se_*, whereas delayed sowing impaired stomatal function despite similar theoretical anatomical capacity (*g_smax_*), revealing a decoupling of structural potential and physiological performance under stress. The adaxial surface consistently exhibited higher *g_s_*, stomatal density, and *g_smax_* than the abaxial surface, highlighting its dominant role in leaf gas exchange. Delayed sowing induced plastic anatomical shifts, including smaller, denser stomata, particularly on the adaxial surface. Significant genotypic variation and moderate heritability were observed for stomatal anatomical traits. We identified 62 putative quantitative trait loci (QTLs) across environments for multiple stomatal traits, including recurring and co-localised loci detected across seasons and times of sowing on chromosome 6B. Pleiotropic QTLs on chromosomes 1A and 2A highlight promising genomic regions. The majority of QTLs (36) were detected for the adaxial surface, positioning it as the dominant surface driving stomatal anatomical variation. A total of 21 QTLs were consistent with chromosomal regions previously reported for stomatal anatomical traits in wheat, particularly on chromosome 7A. In contrast, no QTLs were detected for stomatal physiological traits, indicating limited potential for indirect selection on these traits relative to more stable anatomical traits. Collectively, these findings demonstrate that heat stress uncouples stomatal anatomy from physiological performance in wheat and provides a comprehensive field−based characterisation of stomatal trait integration and its genetic architecture under thermal stress. This work establishes a robust foundation for informing future physiological and genetic studies of wheat heat tolerance, advancing the development of climate-resilient wheat ideotypes.

## Background

Heat stress is a major constraint to global crop productivity, particularly in wheat (*Triticum aestivum* L.), the world’s most widely cultivated cereal (Erenstein et al., 2022). As a staple food for over one-third of the global population (IRDC, 2010) and a cornerstone of Australian agriculture (FAOSTAT, 2024), wheat is especially vulnerable to rising temperatures. Projected global temperature increases of 1.8–5.7 °C by 2100 (OECD, 2012) will intensify the frequency, severity, and duration of heatwaves, particularly in major wheat-growing regions such as the Australian Grain Belt (Anwar et al., 2013; Collins et al., 2024; Perkins-Kirkpatrick and Lewis, 2020). Extreme heat events during critical developmental stages, such as anthesis, cause substantial yield losses and threaten global food security (Pequeno et al., 2021; Ullah et al., 2022; Yashavanthakumar et al., 2021).

Stomata play a central role in balancing photosynthetic carbon assimilation and evaporative cooling (Lawson and Blatt, 2014; Xie et al., 2022). Under heat stress, this balance is disrupted by both stomatal and non-stomatal limitations including damage to photosystem II (PSII), reduced Rubisco carboxylation, impaired ribulose-1,5-bisphosphate (RuBP) regeneration (Chaves and Oliveira, 2004; Chaves et al., 2009), and restricted CO_2_ uptake (Eisenhut et al., 2019; Hodges et al., 2016). These constraints translate into substantial yield penalties, with every 1 °C rise in temperature reducing wheat yields by 6%–10% (Asseng et al., 2015; Helman and Bonfil, 2022; Liu et al., 2016; Shew et al., 2020), and Australian yields are projected to fall by 10%–30% by 2050 (Liu et al., 2016; Potgieter et al., 2013; Zeleke, 2021). Targeting physiological traits that sustain photosynthetic efficiency under heat stress represents a promising avenue to enhance heat resilience.

Stomatal conductance (*g_s_*), the rate at which CO_2_ and water vapour diffuse through the stomata (Wall et al., 2022), not only directly influences photosynthesis and canopy cooling, but also governs water loss (Roche, 2015; Wong et al., 1979). While *g_s_* responds almost instantaneously to environmental cues through physiological adjustments, its operational range is constrained by longer-term anatomical traits, including stomatal size and density (SD; number of stomata per unit leaf area) (Lawson and Leakey, 2024). Wheat shows wide genotypic variation in these traits (Faralli et al., 2024; Lawson and Matthews, 2020; Stevens et al., 2021). As an amphistomatous species, wheat typically exhibits higher adaxial SD, and is the key driver of total leaf *g_s_* (Hõrak, 2025; McAusland et al., 2021; Muir, 2015; Wall et al., 2022). SD is the primary determinant of the maximum potential anatomical stomatal conductance (*g_smax_*), setting the upper physiological limit for gas exchange (Franks et al., 2009; Franks and Beerling, 2009). Integrating anatomical constraints with operating stomatal conductance (*g_sop_*) provides insight into gas exchange efficiency (*g_se_*; *g_se_ = g_sop_/g_smax_*) and stomatal traits associated with enhanced heat tolerance (Busch et al., 2024; Haworth et al., 2021). However, adaxial stomata often realise less of their anatomical *g_smax_* and show greater sensitivity to closure than abaxial stomata, highlighting surface-specific asymmetry in stomatal behaviour (Driesen et al., 2023; Fanourakis et al., 2015; Tulva et al., 2025).

Although responses vary, most studies report reduced *g_s_* under heat stress as a water-conserving response that not only limits transpiration (Balla et al., 2019; Pooja and Munjal, 2019; Redhu et al., 2025), but also restricts CO_2_ uptake (Monneveux et al., 2003). Conversely, several studies report increased *g_s_* under heat when water is not limiting, reflecting either enhanced evaporative cooling (Abdelhakim et al., 2021; Djanaguiraman et al., 2020; Mirosavljević et al., 2021) or inherent heat tolerance in certain germplasm (Abdelhakim et al., 2021; Pinto et al., 2025; Ramya et al., 2016; Sharma et al., 2014). These divergent responses highlight the strong context dependency of stomatal responses to heat, shaped by water availability, evaporative demand, stress duration, and genotypic capacity for thermal regulation. Large genotypic variation in *g*_s_ (Mahdavi et al., 2021; Pooja and Munjal, 2019), heritability estimates of up to 73%, and strong associations between stomatal traits, photosynthetic assimilation, and yield (Li et al., 2021; McAusland et al., 2021; Pinto et al., 2025) highlight significant potential for genetic improvement (Rebetzke et al., 2003, 2001; Romena and Farshadfar, 2019).

Few studies have investigated stomatal anatomical responses to heat stress at breeding-relevant scales, although reduced stomatal pore size and increased SD have been reported in studies with limited genotypes (Fan et al., 2022; Kapadiya et al., 2017; Li et al., 2023). Smaller, denser stomata can open and close more rapidly, facilitating tighter control over gas exchange and evaporative cooling (Drake et al., 2013; McAusland et al., 2016). As with *g_s_*, anatomical responses to heat stress are often stronger adaxially (Bramley et al., 2022; Pinto et al., 2025). Substantial genotypic variation in anatomical traits indicates potential for selection (Condon et al., 2007; McAusland et al., 2021; Pinto et al., 2025), and high heritability and quantitative trait loci (QTLs) have been reported for stomatal anatomical traits, some with pleiotropic effects on yield (Liu et al., 2025; Romena and Farshadfar, 2019; Shahinnia et al., 2016; Wang et al., 2016, 2015). For example, Wang et al. (2015) identified QTL for *g_s_* on chromosomes 2B and 7B, whilst Liu et al. (2025) and Shahinnia et al. (2016) identified loci on chromosomes 4B, 5B, and 7A linked to stomatal size and density, and on chromosome 2B for stomatal size. Ahmed et al. (2021) identified 100 QTLs for stomatal area (SA) and density, across all chromosomes besides 4D, 5D, 6A, and 7D. Despite this, stomatal anatomical traits remain underexplored in breeding programs, particularly under field-based heat stress conditions.

Progress towards integrating stomatal traits into breeding has been limited by a lack of studies linking anatomical potential with in-season physiological performance (Yan, 2021). Coupling measurements of *g_s_* and anatomy can reveal scenarios where there is disconnect between anatomical capacity and realised function, for example, where *g_smax_* is maintained or increased but *g_s_* is reduced (Dow et al., 2014), and can support identification of shared genetic controls across traits whilst mitigating trade-offs with water use efficiency (WUE) (Dwivedi et al., 2021).

Despite their importance in carbon assimilation and WUE (Roche, 2015), progress in scaling precise stomatal measurements to breeding contexts has been limited by methodological constraints. Infrared gas analysers (IRGAs) to measure *g_s_* are accurate but slow and labour-intensive (Guizani et al., 2023; Wang et al., 2024), whilst nail polish imprints to assess stomatal anatomy lack field-suitable scalability (Millstead et al., 2020; Wall et al., 2023, 2022). Recent advances in high-throughput phenotyping, including handheld porometers (e.g., LI-COR LI-600) (McAusland et al., 2023; Reynolds et al., 2020), handheld digital microscopes (Liang et al., 2022; Pathoumthong et al., 2023; Sun et al., 2023, 2021), and deep learning-based image analysis, now provide an opportunity to overcome these constraints in large field trials (Chaplin et al., 2025a; Gibbs and Burgess, 2024).

This study examines the effects of heat stress on *g_s_*, stomatal anatomy, and key physiological indicators such as *g_se_* and *g_smax_* in a diverse panel of 200 wheat genotypes over two seasons, as well as assessing grain yield. We conducted two independent field trials over consecutive seasons: (1) a 2023 trial comparing 200 genotypes and (2) a 2024 trial assessing 50 selected genotypes, both with two time of sowing (TOS) treatments. Heat stress was imposed through delayed sowing (TOS 2), which shifted anthesis into a period of higher ambient temperatures relative to timely sowing (TOS 1). We hypothesised that delayed sowing would expose plants to increased temperatures in the region of 1–3°C at anthesis relative to optimally sown plants. We expected this to reduce *g_sop_*, accompanied by increased SD and reduced stomatal size, with substantial genotypic variation in stomatal traits to support the identification of markers and QTLs for crop improvement. By explicitly linking stomatal anatomy and physiology at breeding-relevant scales under field heat stress, this study addresses a critical gap in our understanding of how stomatal traits interact to confer heat tolerance in wheat.

## Methods

### Plant material, germplasm, and experimental conditions

Field plots were established at the University of Sydney I.A. Watson Grains Research Centre in Narrabri, NSW Australia (30.2743°S, 149.8093°E). Two field trial experiments on wheat (*T. aestivum* L.) were carried out over 2 years (2023 and 2024) to investigate the effect of heat stress, simulated through timely and delayed sowing treatments. In 2023 (season 1; S1), 200 genotypes (**Table 1**) were established with two planting dates; “TOS 1” and “TOS 2”. A randomised complete block design with two field replicates nested within a larger training population was used. To allow more detailed physiological interrogation in 2024 (season 2; S2), a subset of 50 genotypes (**Table 2**) common to S1 were established with two planting dates; TOS 1 and TOS 2. Lines in S2 were selected to maintain connectivity between S1 and S2 and to maximise the diversity of photosynthetic traits across all genotypes. The smaller panel in S2 also allowed for more detailed and replicated interrogation of stomatal traits. In both years, two field replicate plots arranged in randomised blocks were sown for each TOS.

**Table 1:** Performance metrics for the YOLOv8-M model.

Metric	Value
Precision (M)	0.9568
Recall (M)	0.9602
mAP59 (M)	0.9441
mAP50–95 (M)	0.6775

Metrics used were as follows: Precision, Recall, mAP50 (mean average precision at Intersection over Union = 0.5), and mAP50–95 (mean average precision at Intersection over Union from 0.5 to 0.95).

**Table 2:** Linear mixed model ANOVA *p-*values for TOS, variety, and surface on traits of wheat from 2023 field data.

Trait	TOS	Variety	Surface	TOS × variety	TOS × surface	Variety × surface	TOS × variety × surface
Stomatal conductance	<0.001	0.505	<0.001	0.897	<0.001	0.854	0.579
Guard cell width	<0.001	<0.001	<0.001	0.545	0.383	0.183	0.479
Guard cell length	<0.001	<0.001	0.322	0.248	0.002	0.193	0.470
Stomatal area	<0.001	<0.001	0.211	0.260	0.002	0.201	0.505
Stomatal density	<0.001	<0.001	<0.001	0.717	0.174	0.010	0.491
*g_smax_*	0.490	0.0248	<0.001	0.280	0.527	0.015	0.310
*g_se_*	<0.001	0.288	<0.001	0.989	<0.001	0.571	0.650
Yield	<0.001	<0.001	–	<0.001	–	–	–

The diverse germplasm comprised CIMMYT (International Maize and Wheat Improvement Center) and ICARDA (International Centre for Agricultural Research in the Dry Areas) heat-tolerant materials imported through the CIMMYT Australia ICARDA Germplasm Evaluation (CAIGE) programme. Lines include materials from SATYN (Stress Adapted Trait Yield Nursery), EDPIE (Elite Diversity International Experiment), ESWYT (Elite Selection Wheat Yield Trial), SAWYT (Semi-Arid Wheat Yield Trial), HTWYT (High Temperature Wheat Yield Trials), and the University of Sydney including recombinants from three cycles of genomic selection for heat tolerance (including progeny derived from crosses amongst CIMMYT and ICARDA materials).

Plants were sown in 12-m^2^ plots (2 × 6 m) with five planting rows at a planting density of 100 plants/m^2.^ Plots were subsequently trimmed 8 m^2^ (2 × 4 m) before harvest. The distance between rows in each plot (row spacing) was approximately 23.5 cm and the distance between each plot was 63 cm. The predominant soil type at the field site is a black vertosol cracking clay with high water retention. For both trials, minimum tillage was used to maintain soil integrity and moisture. Field traffic was controlled as much as possible with allocated roads and pathways and GPS guidance on all machinery. Fertiliser application was kept consistent across both treatments and both trials [Urea (46% N) 100 kg/ha and Cotton Sustain (5% N, 10% P, 21% K, 1% Z) 80 kg/ha pre-planting]. The experimental sites were fallowed over the summer months and rotated with a legume crop during alternate years to minimise disease outbreak and maintain soil integrity.

In S1, TOS 1 was planted on 30 May 2023 and harvested on 31 October 2023 whilst TOS 2 was planted on 19 July 2023 and harvested on 13 December 2023. At planting, average soil moisture was 18.8% at 10 cm, 19.5% at 30 cm, 17.3% at 50 cm, and 19.9% at 100 cm at TOS 1 and 31.6%, 36.8%, 34.1%, and 36.6% at TOS 2 at 10, 30, 50, and 100 cm, respectively. Irrigation was used to limit the confounding effects of moisture stress, particularly at TOS 2. TOS 1 plants received 150 mm of rainfall and irrigation whilst TOS 2 received 227.9 mm of total moisture from rainfall and irrigation. The mean daily minimum and maximum temperature across the growing season were 6.1°C and 22.8°C for TOS 1, respectively, and 9.7°C and 27.2°C for TOS 2, respectively. During anthesis measurement windows, mean daily minimum and maximum temperatures were 2.7°C and 24.2°C at TOS 1, and 10.1°C and 27.1°C at TOS 2. For the same period, relative humidity (RH) ranged from 25.0% to 84.3% at TOS 1 and 30.8%–84.8% at TOS 2, with corresponding mean vapour pressure deficit (VPD) of 0.70 and 0.90 kPa, respectively.

In S2, TOS 1 was planted on 22 May 2024 and harvested on 6 November 2024, and TOS 2 was planted on 23 July 2024 and harvested on 27 November 2024. At planting, average soil moisture was 18.2% at 10 cm, 15.6% at 30 cm, 14.6% at 50 cm, and 11.1% at 100 cm at TOS 1 and 28.0%, 31.4%, 31.7%, and 31.0% at TOS 2 at 10, 30, 50, and 100 cm, respectively. Irrigation was used to limit the confounding effects of moisture stress, particularly at TOS 2. The total moisture from rainfall and irrigation for TOS 1 and TOS 2 was 278.4 and 160.4 mm, respectively. The mean daily minimum and maximum temperature across the growing season were 6.4°C and 21.2°C for TOS 1, respectively, and 8.8°C and 25.1°C for TOS 2, respectively. During measurements at anthesis, mean daily minimum and maximum temperatures were 4.4°C and 22.0°C at TOS 1, and 9.7°C and 26.7°C at TOS 2. RH for the measurement period ranged from 34.5% to 92.0% at TOS 1 and from 38.5% to 91.0% at TOS 2, with corresponding mean VPD of 0.56 and 0.74 kPa, respectively.

### Field measurements

In S1 and S2, measurements of stomatal physiology and anatomy were taken from fully expanded flag leaves at anthesis (Zadok stages 48–68) and chosen using a systematic randomised sampling technique with representative plants selected from the middle three planting rows of each plot, at least 50 cm from the end of each plot. In all seasons, data were collected on consecutive days, and all measurements were taken between 09:30 and 14:00 to minimise diurnal effects on stomatal conductance and rates of photosynthesis. In S1, two leaves were sampled from each replicate plot per TOS (*n* = 4 per genotype per TOS). In S2, three leaves were sampled from each replicate plot per TOS (*n* = 6 per genotype per TOS), reflecting the smaller number of lines and implementation of a streamlined high-throughput phenotyping approach that increased sampling capacity in S2 (Chaplin et al., 2025a). Zadok scores were taken at each plot during measurement of traits for inclusion in statistical models.

#### Stomatal conductance

In S1 and S2, a LI-COR LI-600 porometer/fluorometer (Li-COR Inc., Nebraska, USA) was used to measure *g_s_* of both leaf surfaces (adaxial and abaxial) (**Supplementary Figure 1**). The settings were as follows: flow rate: 150 µmol s^−1^, phase length: 300 ms, ramp amount: 25%, leaf absorptance: 0.8, fraction abs PSII: 0.5, actinic modulation rate: 500 Hz, and integrated modulation intensity: 6.67 µmol m^−2^ s^−1^, stability: medium. Measurements were collected from the middle portion of the leaf blade from the flag leaf of the main tiller in full sunlight, as described by Chaplin et al. (2025a).

#### Stomatal anatomy image capture

In S1 and S2, in-field microscopy imaging of the abaxial and adaxial surfaces of the same leaf was carried out. In S1, a 200× magnification handheld USB microscope was used (Dino-Lite 5MP, AM7515MT2A; Dino-Lite, AnMo Electronics Corporation, Taiwan) and in S2, a 400× magnification model was used (Dino-Lite 5MP, AM7515MT4A; Dino-Lite, AnMo Electronics Corporation, Taiwan). In both seasons, the microscope was configured with LED brightness = 5, LED axial light = 0 and resolution = 2,592 × 1,944.

To aid focussing of the microscope in the field, a custom-designed 3D-printed leaf clip with a smaller aperture than the cap provided with the microscope was used. Black electrical tape was wrapped around the clear plastic part of the microscope shaft to ensure consistent lighting conditions and prevent external light from hitting the leaf surface. The microscope was connected by a USB cable to a Windows computer (Dell Latitude 7230 Rugged Extreme Tablet). Each leaf was imaged by clamping the leaf into the leaf clip, on the same portion of leaf measured with the porometer. Fine focusing on the leaf surface was achieved by adjusting pressure on the trigger with the leaf clamped in the leaf clip. In S2, following image capture, leaves were then excised and placed in an envelope for subsequent nutrient component analysis. Images were saved using a custom image capture app, FieldDino, which was built with Python and PyQt5, and using the Dino-Lite SDK. The app and guidance on its installation are available on the Github repository (https://github.com/williamtsalter/FieldDinoMicroscopy). As described by Chaplin et al. (2025a), it incorporates key elements of field data collection:

1. Pre-filled image names based on an existing spreadsheet to ensure each image matches the plot in which it is collected
2. Clear video feed with recordable and adjustable microscope parameters
3. Easy-to-use buttons and image deletion

#### Yield and yield components

At maturity, plots were machine-harvested and grain yield was recorded and expressed on a per-hectare basis. In S2, subsamples of cleaned grain were analysed for quality traits. Thousand kernel weight (TKW) was determined using an optical seed counter (Contador, Pfeuffer GmbH, Germany), with results expressed on a dry-weight basis. Screenings (%) were quantified by passing grain over a standard 2.8-mm slotted sieve and calculating the proportion of grain retained versus discarded. Grain protein content, test weight, and moisture content were measured using a near-infrared spectroscopy instrument (FOSS analytical system) following manufacturer guidelines.

### Data analyses

#### Stomatal anatomy and deep learning model

In all seasons, stomatal traits were quantified using a deep-learning-based image analysis pipeline, as described by Chaplin et al. (2025a). In brief, 121 images were initially annotated via instance segmentation using Roboflow with the images split into train (60%), validation (30%), and test (10%). Each image was then split into 1,280 × 1,280 tiles and exported as both an original and augmented image. Image augmentations included random rotations, flips, contrast, and saturation changes. Tiling and augmentation resulted in 822 train images, 420 validation images, and 78 test images. YOLOv8 models of varying sizes were trained and evaluated. YOLOv8-M, which ran for 119 epochs, was selected for further analyses based on performance metrics including precision, recall, and mean average precision (mAP) performance (**Table 1**). Stomatal traits including SD and guard cell dimensions were extracted using a custom Python script employing Ultralytics and OpenCV. This performed automated stomatal detection, and a fitted ellipse around each stomata enabled anatomical measurements to be estimated (**Supplementary Figures S2, S3**). Guard cell length (GCL) was defined as the major axis of the fitted ellipse, representing the longitudinal dimension of the stomatal pore complex. Guard cell width (GCW) was defined as the minor axis of the fitted ellipse, representing the transverse dimension. SA was calculated based on the pixel area of detected stomata, providing an integrative anatomical metric, and *g_smax_* was calculated according to Franks and Beerling (2009). To refine *g_smax_* calculations, the conventional assumption that pore length is half GCL was tested (Franks and Beerling, 2009; Franks and Farquhar, 2007). The empirically determined value (0.543) was incorporated into *g_smax_* calculations. Full methodological details for stomatal anatomy image analysis are provided in Chaplin et al. (2025a) and are available via the GitHub repository: https://github.com/williamtsalter/FieldDinoMicroscopy.

#### Phenotypic visual and statistical analyses

Phenotypic data visualisation was performed with R (R Core Team, 2021) using packages dplyr (Wickham et al., 2023), ggplot2 (Wickham, 2016), gridExtra (Baptiste, 2017), and tidyr (Wickham et al., 2024). Statistical analyses were performed with R (R Core Team, 2021) using a linear mixed-effects model (LMM) to account for potential variation amongst replicates. The model was fitted using the lme4 package in R, with Treatment, Variety, and Surface as fixed effects, and Rep and Leaf as random effects:


$$Response\;Var\;(\text{e}.\text{g}.,g_{s})\sim Treatment\times Variety\times Surface+(1|Rep)+(1|Leaf)$$

Analysis of variance (ANOVA) was performed to assess the significance of fixed effects and their interactions, and *post-hoc* pairwise comparisons were conducted using the emmeans package, with Tukey’s adjustment for multiple comparisons between separate treatment groups. Packages lme4 (Bates et al., 2015), lmerTest (Kuznetsova et al., 2017), ggplot2 (Wickham, 2016), emmeans (Lenth et al., 2020), and MASS (Venables and Ripley, 2002) were used for analyses.

Prior to model fitting, assumptions of normality and homoscedasticity of residuals were assessed visually. Where assumptions were not met, the Box–Cox transformation (Box and Cox, 1964) was applied using the boxcox function from the MASS package. The optimal transformation parameter, λ, was identified by maximising the log-likelihood of a fixed-effects linear model across a range of λ ϵ [−2,2]. Based on the estimated λ, the most appropriate transformation was applied (e.g., log if λ≈0, square root if λ≈0.5, cube root if λ≈0.33) prior to fitting the mixed-effects model (Wilkinson and Rogers, 1973). Interpretation of the TOS effect should be made with caution, as it may be partially attributable to spatial variation resulting from TOS being spatially confounded with block.

#### Genotypic visual and statistical analyses

The materials were genotyped using the Illumina Infinium Wheat Barley 40K SNP array (Keeble-Gagnère et al., 2021). The array contains 25,363 wheat-specific and 14,261 barley-specific SNP markers and enables accurate imputation of SNP data. It can therefore be used to infer genotypes at untyped chromosomal locations for downstream QTL analysis.

Prior to the QTL analysis, broad-sense heritability was estimated for each trait, trial, and surface from the phenotypic model using the approach by Cullis et al. (2006) via the heritable package (Kar et al., 2026). More specifically, an appropriately transformed phenotypic trait was modelled using linear mixed models that included variety and experimental design factors (Row, Range, Plot, and Sampling Date) as random effects assuming independent Normal distribution with mean zero and constant variance using the asreml R package (Butler et al., 2023).

If the estimated heritability was greater than 0.05, then putative QTLs were searched using a whole genome approach using the wgaim package (Taylor and Verbyla, 2011) with population structure correction made by fitting leading projected principal components (PCs) of the column-centred genotype markers, where rows are the genotypes and column are the markers, as fixed effects in the working model (Price et al., 2006; Yu et al., 2006). The number of PCs was selected using the elbow method from the visual inspection of the scree plot of the eigenvalues (chosen as nine, see **Supplementary Figure 4**). The wgaim method has been previously applied in Taylor et al. (2023) for finding QTLs for a spot in a diverse population of wheat where population structure correction was made with known subpopulations. Because wgaim relies on asreml to fit the linear mixed model, it offers substantial flexibility in the model specification. As the experimental design consisted of two levels of replication (plot level and leaf level), it necessitates linear mixed models that simultaneously account for both plot-to-plot and leaf-to-leaf variation, thus allowing for better separation of noise from signal. Not accounting for these two variations will result in pseudo-replication (Hurlbert, 1984).

The wgaim approach involves iteratively fitting each marker as fixed effects to a modified phenotypic model in a forwards selection procedure to select significant markers, whilst accounting for background level of genetic variation through random additive marker effects using the genomic relationship matrix (Verbyla et al., 2007). More specifically, the working model in wgaim is a linear mixed model,


$$y=X\beta +Z_{g}u_{a}+Z_{g}u_{p}+Zu+e$$

where y is a vector of appropriately transformed phenotypic trait, X is the design matrix for fixed effects β  (in this case, the overall mean and the nine selected PCs), $Z_{g}$ is the design matrix that maps the genotype to observation, $u_{a}\sim N(0,\;\sigma_{a}^{2}K)$ is a vector of random additive variety effects where K is the genomic relationship matrix, $u_{p}\sim N(0,\;\sigma_{p}^{2}I)$ is a vector of random non-additive polygenic effects, Z is the design matrix of non-genetic random effects u associated with experimental design factors (Row, Range, Plot, and Sampling Date) where the ith factor is assumed to be $N(0,\;\sigma_{i}^{2}I)$, and $e\sim N(0,\;\sigma^{2}I)$is a vector of residuals. The marker was considered significant using the default family-wise error rate of 0.05 and using an exclusion window of 10 mega-base pair (Mbp). These significant markers are added progressively to fixed effects. The full analysis code for genome-wide association study (GWAS) is available via the data repository provided.

## Results

### Season 1—200 genotypes

#### Stomatal conductance

**Figure 1:**
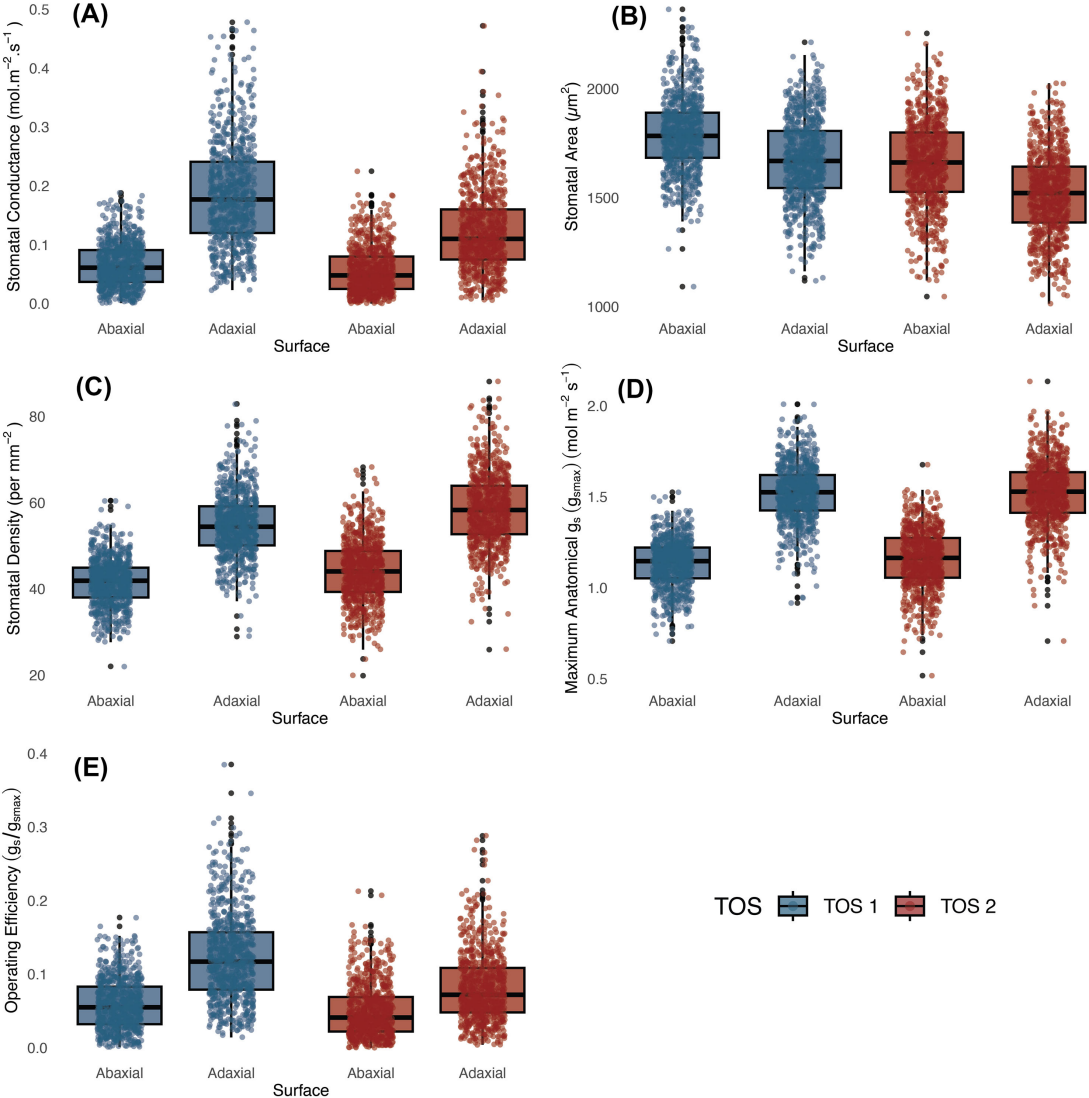
Operational stomatal conductance (*g*_sop_) and anatomical traits across 200 wheat genotypes at two sowing times in season 1. **(a)** *g*_sop_; **(b)** stomatal area; **(c)** stomatal density; **(d)** maximal anatomical stomatal conductance; and **(e)** stomatal conductance operating efficiency (*g_se_*). Boxes represent the interquartile range (25th–75th percentiles), with horizontal lines indicating the median. Whiskers denote the minimum and maximum values, and points represent individual observations.

In S1, *g*_s_ was significantly affected by TOS, leaf surface, and their interaction (*p* < 0.001) (**Table 2**). Plants sown earlier (TOS 1) had higher *g*_s_ than those sown later (TOS 2) for both surfaces (abaxial *p* < 0.01; adaxial *p* < 0.001), with the highest values on the adaxial surface at TOS 1 (**Figure 1A**). On average, *g*_s_ declined by 28.9% at TOS 2 (**Supplementary Table 3**). Leaf surface had the strongest effect (*p* < 0.001), with adaxial *g*_s_ consistently exceeding abaxial *g*_s_ (*p* < 0.001). A significant TOS × surface interaction (*p* < 0.001) indicated that TOS influenced surfaces differently; abaxial *g*_s_ declined by 14.8% from TOS1 to TOS2, whilst adaxial *g*_s_ dropped by 33.9% (**Supplementary Table 3**), making the adaxial surface the main contributor to overall gas exchange and the primary driver of reductions under warmer TOS 2 conditions. No significant genotypic variation was detected amongst the 200 genotypes.

#### Stomatal anatomy

GCL was significantly larger at TOS 1 than TOS 2 (*p* < 0.001) (**Table 2**), with strong genotype effects (*p* < 0.001). A significant TOS × surface interaction (*p* < 0.01) indicated that TOS influenced GCL differently across leaf surfaces. *Post-hoc* tests showed adaxial GCL exceeded abaxial only at TOS 1 (*p* < 0.001). Mean GCL declined by 5.0% from TOS 1 to TOS 2 (**Supplementary Table 3**).

GCW was also significantly larger at TOS 1 than TOS 2 (*p* < 0.001), averaging 3.0% lower in TOS 2 (**Supplementary Table 3**). Surface had a strong effect (*p* < 0.001), with abaxial GCW consistently larger than adaxial (*p* < 0.001). Genotype also influenced GCW (*p* < 0.001), but no significant interactions amongst TOS, surface, and genotype were detected (all *p* > 0.17), indicating independent effects.

SA was also significantly larger at TOS 1 than TOS 2 (*p* < 0.001), declining by 8.2% (**Figure 1B**; **Supplementary Table 3**). Genotype effects were significant (*p* < 0.001), whilst surface showed no main effect (*p* = 0.211) (**Table 2**). A TOS × surface interaction (*p* < 0.01) indicated greater reductions on the adaxial surface between TOS 1 and TOS2 compared to the abaxial surface.

**Figure 2:**
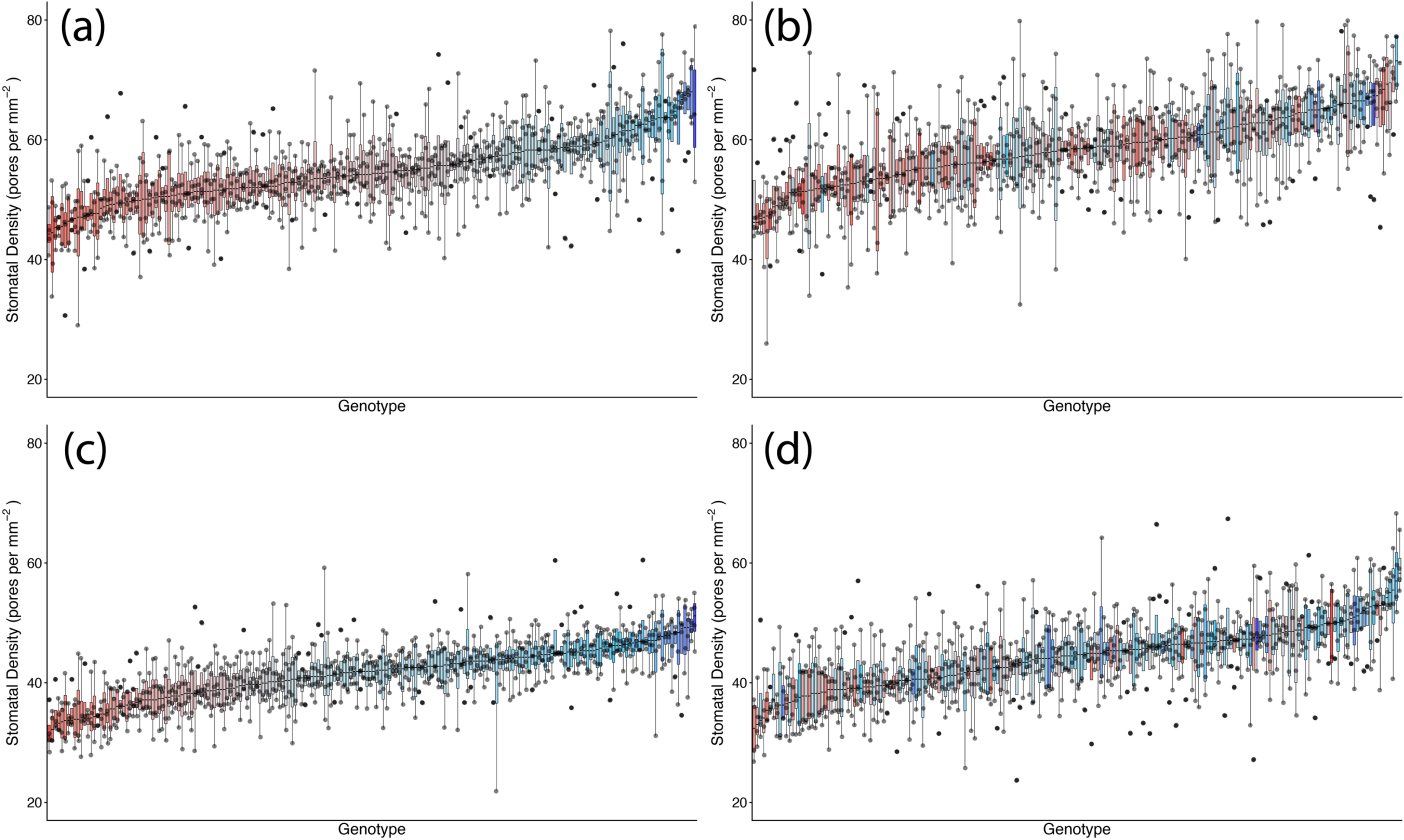
Genotypic distribution of stomatal density across 200 wheat genotypes at two sowing times in season 1. **(a)** Adaxial surface TOS 1; **(b)** adaxial surface TOS 2; **(c)** abaxial surface TOS 1; and **(d)** abaxial surface TOS 2. Genotypes are ranked by median stomatal density. Colour assigned to each genotype based on TOS 1 stomatal density. Thick horizontal lines within boxes indicate the median and boxes indicate the upper (75%) and lower (25%) quartiles. Whiskers indicate the ranges of the minimum and maximum values. Points indicate individual measurements.

Stomatal density was significantly affected by TOS, genotype, and surface (all *p* < 0.001) (**Table 2**; **Figures 1C**, **2**). A significant genotype × surface interaction (*p* < 0.01) indicated that genotypic effects differed between surfaces, whereas TOS × variety (*p* = 0.717) and TOS × surface (*p* = 0.174) interactions were not significant, suggesting that TOS effects were consistent across genotypes and surfaces. *Post-hoc* tests confirmed that SD was larger at TOS 2 than TOS 1 for both surfaces (*p* < 0.001), increasing by 6.4% (abaxial) and 6.7% (adaxial) (**Supplementary Table 3**). Adaxial SD exceeded abaxial across treatments (*p* < 0.001).

SD correlated positively with *g*_s_ (*R*² = 0.1383) and negatively with SA (*R*² = 0.321) (**Supplementary Figure 5**).

**Figure 3:**
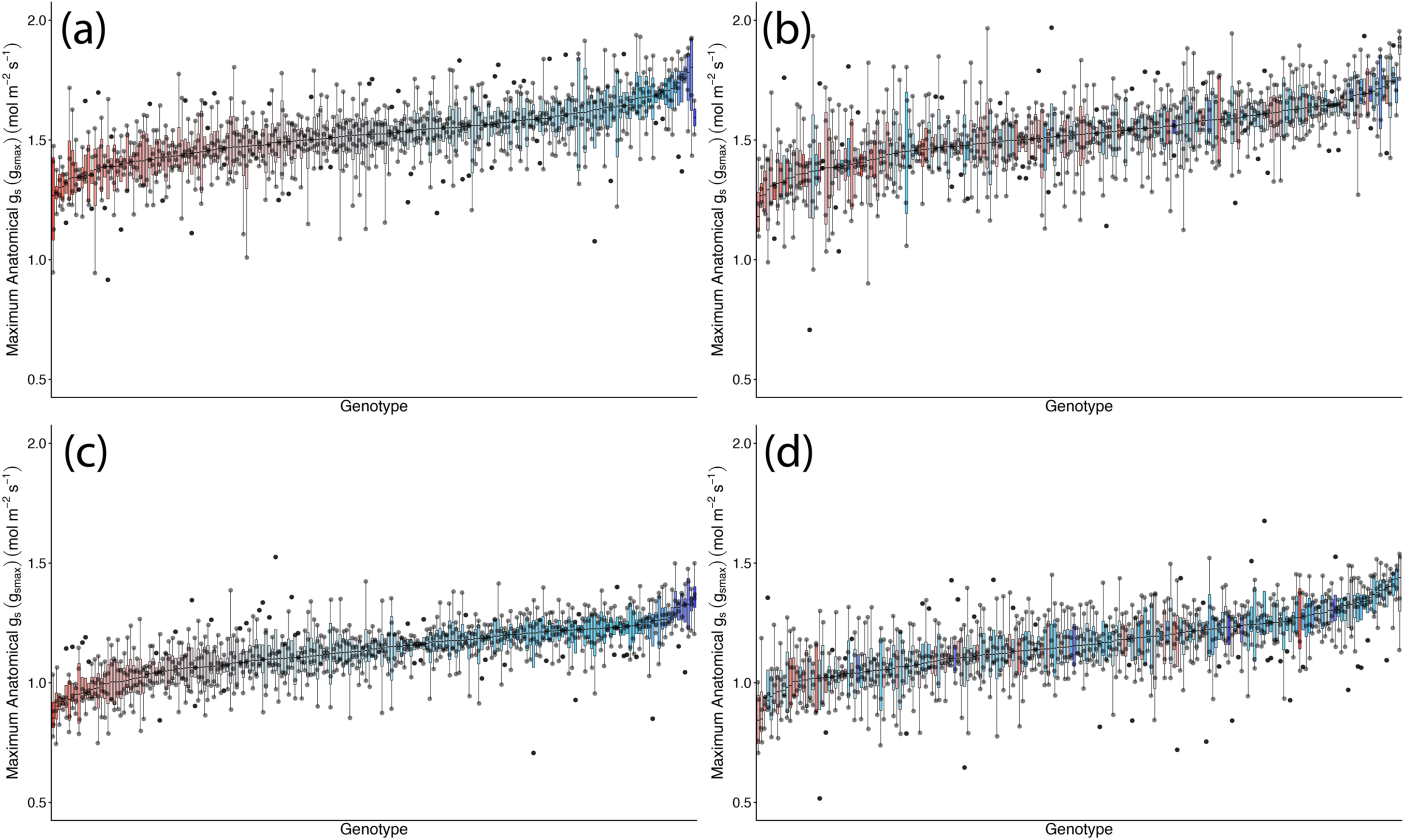
Genotypic distribution of maximum anatomical stomatal conductance (*g_smax_*) across 200 wheat genotypes at two sowing times in season 1. **(a)** Adaxial surface TOS 1; **(b)** adaxial surface TOS 2; **(c)** abaxial surface TOS 1; and **(d)** abaxial surface TOS 2. Genotypes are ranked by median *g_smax_*. Colour assigned to each genotype based on TOS 1 *g_smax_*. Thick horizontal lines within boxes indicate the median and boxes indicate the upper (75%) and lower (25%) quartiles. Whiskers indicate the ranges of the minimum and maximum values. Points indicate individual measurements.

*g*_smax_, estimated from stomatal anatomy, showed no significant effect of TOS but a weak genotypic effect (*p* < 0.05), indicating differences amongst genotypes. Median adaxial *g*_smax_ ranged from 1.27 to 1.84 mol m^−2^ s^−1^ (TOS 1) and from 1.18 to 1.91 mol m^−2^ s^−1^ (TOS 2); abaxial values ranged from 0.85 to 1.36 mol m^−2^ s^−1^ and from 0.84 to 1.44 mol m^−2^ s^−1^, respectively (**Table 2**, **Figure 3**). Surface had a strong effect (*p* < 0.001) on *g*_smax_, with adaxial estimates consistently higher than abaxial across TOS (*p* < 0.001) (**Figure 1D**). A significant variety × surface interaction (*p* < 0.05) indicated that genotype effects on *g*_smax_ differed between leaf surfaces. No three-way interaction was detected (*p* = 0.310). *g*_smax_ was positively correlated with measured *g*_s_ (*R*² = 0.213).

#### Integrated stomatal conductance and anatomy

When *g*_s_ and *g*_smax_ were combined to calculate *g*_se_ (unitless), significant effects of TOS and surface were detected (*p* < 0.001) (**Table 2**, **Figure 1E**), along with a strong TOS × surface interaction (*p* < 0.001), indicating that TOS effects varied by leaf surface. *Post-hoc* tests revealed that *g*_se_ was higher at TOS 1 than TOS 2 for both surfaces (*p* < 0.001), declining by 15.2% on the abaxial and 33.3% on the adaxial surface (**Supplementary Table 3**). Across both TOS, adaxial *g*_se_ exceeded abaxial *g*_se_ (*p* < 0.001). There was no significant variation across genotypes.

#### Yield parameters

##### Grain yield

Grain yield was significantly higher at TOS 1 compared with at TOS 2 (*p* < 0.001) (**Table 2**). Mean grain yield at TOS 1 was 4.62 t/ha, whilst the mean grain yield at TOS 2 was 2.79 t/ha, equating to a 39.6% reduction between TOS. Genotypes also varied significantly with regard to grain yield (*p* < 0.001). Additionally, the TOS × Genotype interaction was significant (*p* < 0.001), suggesting that the effect of TOS on yield varies dependent on genotype. No significant relationships were found between yield and stomatal traits.

#### Genome–phenome analyses

Stomatal anatomical traits (GCL, GCW, SA, and SD) exhibited heritability estimates of 0.375 to 0.677, whilst the heritability estimates for stomatal conductance traits (*g*_s_ and *g_se_*) were generally lower, ranging from 0.094 to 0.392, although *g_smax_* was higher, ranging from 0.440 to 0.653 (**Table 3**). While there were no considerable differences in estimated heritability across TOS for abaxial surfaces, all heritability estimates were lower at TOS 2 for the adaxial surface.

**Table 3:** Broad-sense heritability estimates for each trait, arranged by surface, TOS and year.

Trait	Abaxial				Adaxial			
	TOS1		TOS2		TOS1		TOS2	
	2023	2024	2023	2024	2023	2024	2023	2024
Stomatal conductance	0.392	0.252	0.150	0.000	0.229	0.000	0.153	0.000
Guard cell width	0.566	0.632	0.571	0.587	0.578	0.613	0.477	0.335
Guard cell length	0.609	0.763	0.572	0.610	0.617	0.720	0.375	0.621
Stomatal area	0.574	0.691	0.633	0.570	0.656	0.625	0.449	0.548
Stomatal density	0.677	0.618	0.563	0.680	0.527	0.296	0.426	0.545
*g_smax_*	0.653	0.470	0.601	0.658	0.487	0.048	0.440	0.411
*g_se_*	0.390	0.088	0.112	0.000	0.235	0.000	0.094	0.000

**Figure 4:**
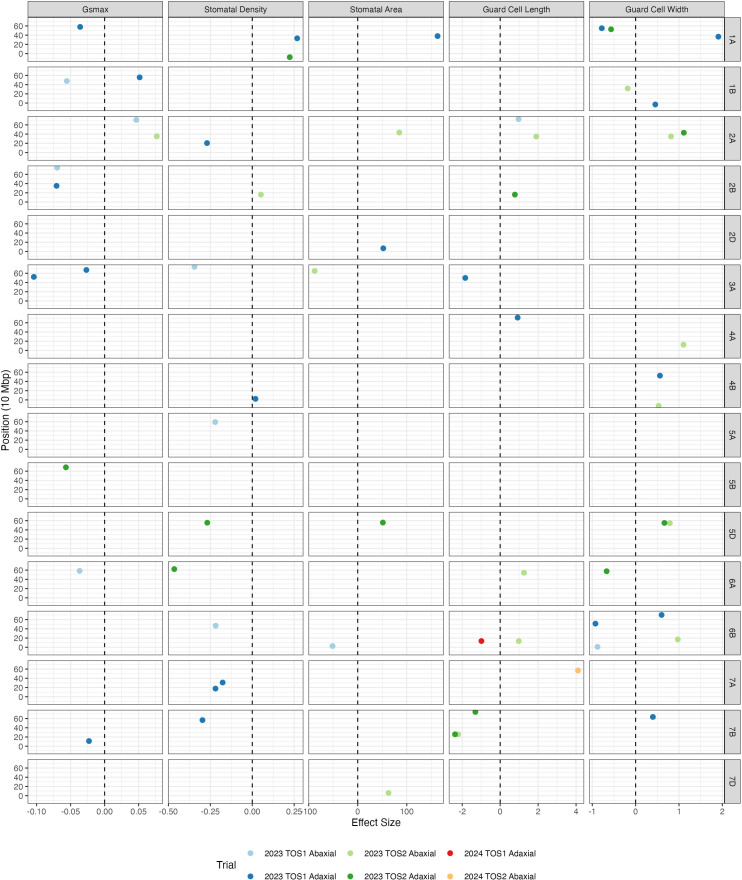
Effective sizes of putative QTLs by trait and chromosome. The scatterplot shows the effect sizes of putatiative QTLs identified by chromosome and position (in mega-base pairs), with each colour corresponding to a specific year, TOS, and surface. The vertical dashed lines are the *y*-intercepts. The points are slightly jittered so the points are visible.

At least one putative QTL candidate was identified for each anatomical trait for every surface and TOS, except *g_smax_* for the adaxial surface at TOS 2 (**Table 4**). In total, 60 putative QTLs (25 abaxial and 35 adaxial) were found across stomatal anatomical traits. All traits appear to be explained by several chromosomal locations across the whole genome (**Figure 4**; **Supplementary Figure 6**; **Supplementary Table 4**). One marker had noticeably larger LOD scores than others at chromosome 1A for *g_smax_* (LOD = 8.8) (**Supplementary Figure 6**). Notably, chromosomes 1A, 3A, 6A, 6B, and 7B appear to contain several QTLs across four stomatal anatomical traits whilst chromosome 2A contains a high number of QTLs across all five traits (**Supplementary Table 4**; see the full list of putative QTLs in **Supplementary Table 5**).

**Table 4:** The number of putative QTL candidates by year, TOS, and surface for each trait. The “.” represents 0.

Trait	Abaxial		Adaxial		Abaxial	Adaxial
	2023				2024	
	TOS1	TOS2	TOS1	TOS2	TOS2	TOS1
Guard cell width	1	6	7	4	.	.
Guard cell length	1	4	2	3	1	1
Stomatal area	1	3	2	1	.	.
Stomatal density	3	1	6	3	.	.
*g_smax_*	4	1	6	1	.	.

### Season 2—50 genotypes

#### Stomatal conductance

**Figure 5:**
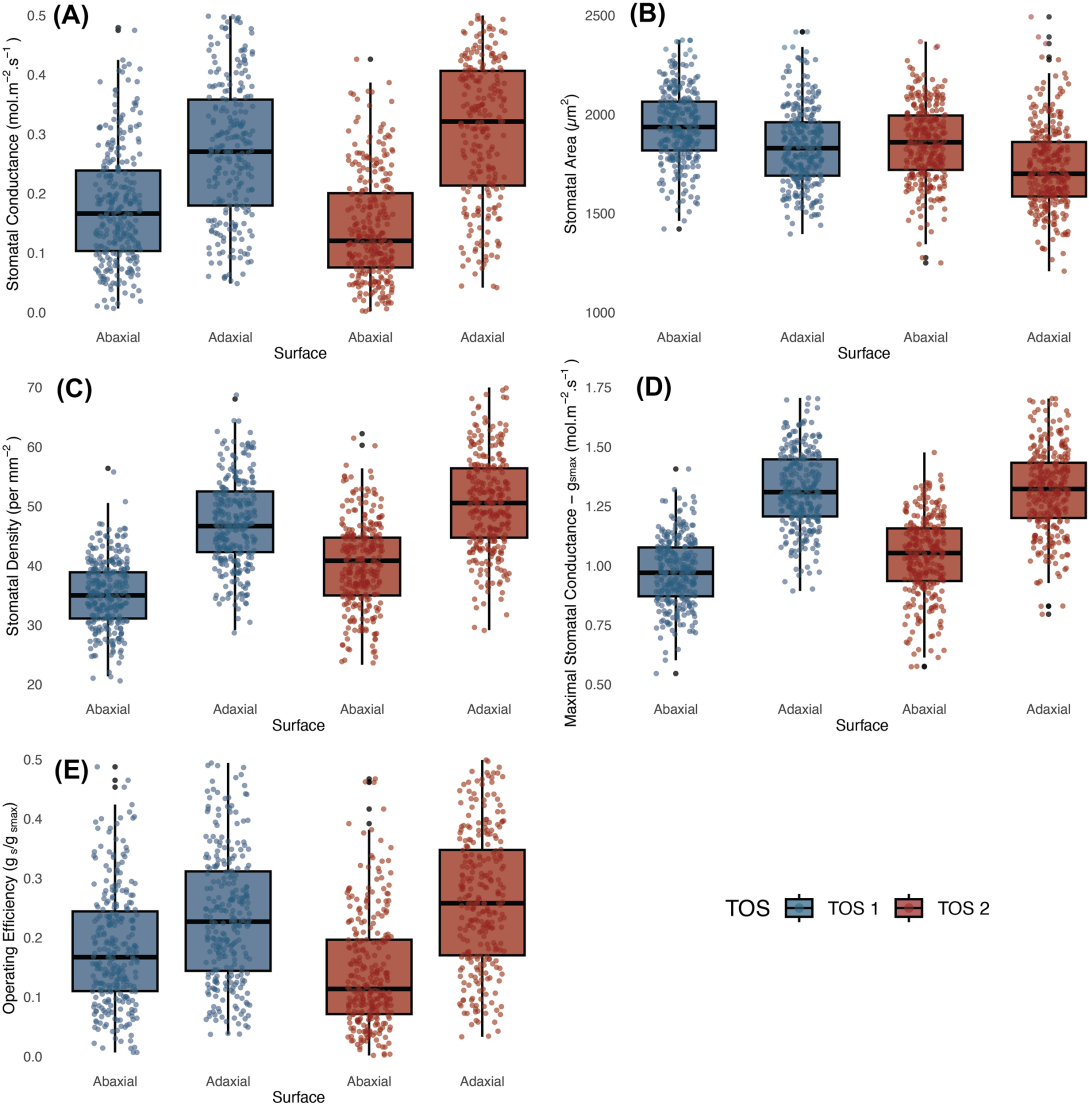
Operational stomatal conductance (*g*_sop_) and anatomical traits across 200 wheat genotypes at two sowing times in season 2. **(a)** *g*_sop_; **(b)** stomatal area; **(c)** stomatal density; **(d)** maximal anatomical stomatal conductance; and **(e)** stomatal conductance operating efficiency (*g_se_*). Boxes represent the interquartile range (25th–75th percentiles), with horizontal lines indicating the median. Whiskers denote the minimum and maximum values, and points represent individual observations.

**Figure 6:**
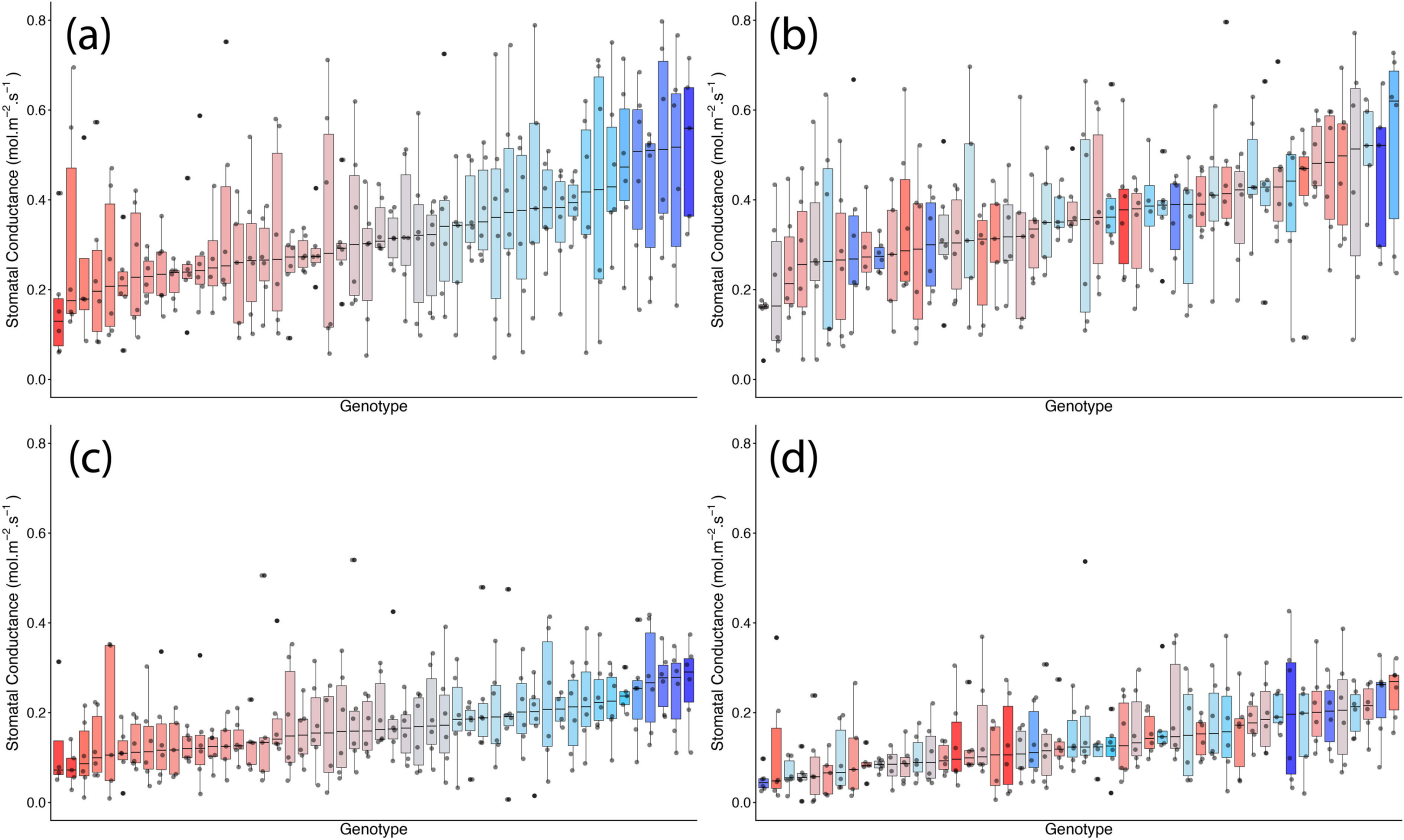
Genotypic distribution of stomatal conductance (*g_s_*) across 50 wheat genotypes at two sowing times in season 2. **(A)** Adaxial surface TOS 1; **(B)** adaxial surface TOS 2; **(C)** abaxial surface TOS 1; and **(D)** abaxial surface TOS 2. Genotypes are ranked by median *g_s_*. Colour assigned to each genotype based on TOS 1 *g_s_*. Thick horizontal lines within boxes indicate the median and boxes indicate the upper (75%) and lower (25%) quartiles. Whiskers indicate the ranges of the minimum and maximum values. Points indicate individual measurements.

In S2, *g*_s_ was significantly affected by genotype and leaf surface (both *p* < 0.001), along with several interactions (**Table 5**). However, the LMM showed no main effect of TOS (*p* = 0.325) (**Figure 5A**). Leaf surface exerted the strongest influence on *g*_s_ (*p* < 0.001), with adaxial *g_s_* consistently higher than abaxial *g_s_* (*p* < 0.001). Genotypic variation was substantial (*p* < 0.001) amongst the 50 wheat genotypes (**Figure 6**). Median ranges with median adaxial *g*_s_ ranging from 0.130 to 0.605 mmol m^−2^ s^−1^ (TOS 1) and from 0.162 to 0.620 mmol m^−2^ s^−1^ (TOS 2) and abaxial *g*_s_ from 0.072 to 0.291 mmol m^−2^ s^−1^ (TOS 1) and from 0.044 to 0.270 mmol m^−2^ s^−1^ (TOS 2).

**Table 5:** Linear mixed model ANOVA *p-*values for TOS, variety, and surface on traits of wheat from 2024 field data.

Trait	TOS	Variety	Surface	TOS × variety	TOS × surface	Variety × surface	TOS × variety × surface
Stomatal conductance	0.325	<0.001	<0.001	<0.001	<0.001	0.181	0.871
Guard cell width	0.267	<0.001	<0.001	<0.001	0.988	0.005	0.204
Guard cell length	<0.001	<0.001	<0.001	<0.001	0.015	0.152	0.720
Stomatal area	<0.001	<0.001	<0.001	<0.001	0.166	0.369	0.625
Stomatal density	<0.001	<0.001	<0.001	<0.001	0.011	0.014	0.143
*g_smax_*	<0.001	<0.001	<0.001	0.018	<0.001	<0.001	0.196
*g_se_*	0.123	<0.001	<0.001	<0.001	<0.001	0.052	0.300
Yield	<0.001	<0.001	–	<0.001	–	–	–
Thousand kernel weight	<0.001	<0.001	–	<0.001	–	–	–
Screenings %	<0.001	<0.001	–	<0.001	–	–	–
Protein	<0.001	<0.001	–	<0.001	–	–	–
Moisture	<0.001	<0.001	–	<0.001	–	–	–
Test weight	<0.001	<0.001	–	<0.001	–	–	–

Although TOS had no overall effect, a significant TOS × genotype interaction (*p* < 0.001) indicated non-uniform genotypic responses, with some lines showing greater declines at TOS 2. Similarly, a significant TOS × surface interaction (*p* < 0.001) revealed that TOS influenced surfaces differently, with *post-hoc* tests confirming a stronger TOS effect on adaxial *g*_s_.

#### Stomatal anatomy

GCL varied significantly amongst genotypes (*p* < 0.001), between TOS (*p* < 0.001), and across leaf surfaces (*p* < 0.001) (**Table 5**). *Post-hoc* tests showed that GCL was higher at TOS 1 than TOS 2 for both surfaces (*p* < 0.001). At TOS 1, adaxial GCL exceeded abaxial GCL (*p* < 0.001), whilst no surface difference was detected at TOS 2 (*p* = 0.167). Significant TOS × variety (*p* < 0.001) and TOS × surface (*p* < 0.05) interactions indicate that TOS effects varied by genotype and surface.

GCW was significantly affected by leaf surface and genotype (both *p* < 0.001) but not by TOS (**Table 5**). *Post-hoc* tests confirmed that GCW was consistently higher on the abaxial surface than the adaxial surface at both TOS (*p* < 0.001). Significant TOS × genotype (*p* < 0.001) and genotype × surface (*p* < 0.01) interactions indicate that TOS effects varied amongst genotypes and that surface contributed to genotypic differences in GCW.

SA was significantly affected by TOS and leaf surface (both *p* < 0.001) (**Table 5**, **Figure 5B**), with strong genotypic variation (*p* < 0.001). SA ranged from 1,421 to 2,602 µm^2^ (abaxial) and from 1,396 to 2,661 µm^2^ (adaxial) at TOS 1, and from 1,250 to 2,367 µm^2^ (abaxial) and from 1,208 to 2,493 µm^2^ (adaxial) at TOS 2. *Post-hoc* tests confirmed that SA was higher on the abaxial surface than on the adaxial surface at both TOS (*p* < 0.001) and higher at TOS 1 than at TOS 2 for both surfaces (*p* < 0.001), with a 4.7% decline on the abaxial surface and 6.2% on the adaxial surface between TOS 1 and TOS 2 (**Supplementary Table 6**). A significant TOS × variety interaction (*p* < 0.001) indicates genotype-dependent responses to TOS.

**Figure 7:**
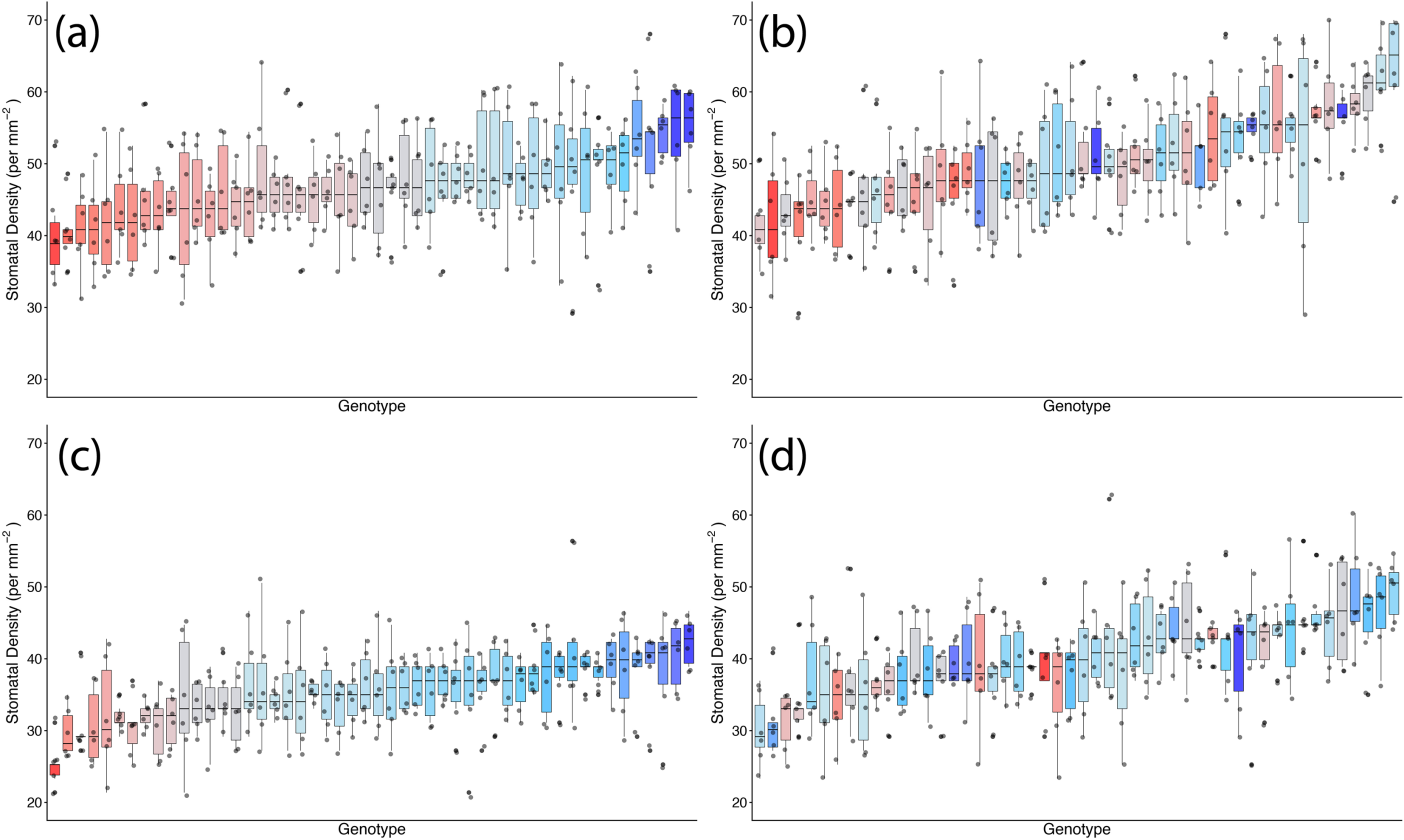
Genotypic distribution of stomatal density across 50 wheat genotypes at two sowing times in season 2. **(A)** Adaxial surface TOS 1; **(B)** adaxial surface TOS 2; **(C)** abaxial surface TOS 1; and **(D)** abaxial surface TOS 2. Genotypes are ranked by median stomatal density. Colour assigned to each genotype based on TOS 1 stomatal density. Thick horizontal lines within boxes indicate the median and boxes indicate the upper (75%) and lower (25%) quartiles. Whiskers indicate the ranges of the minimum and maximum values. Points indicate individual measurements.

SD was significantly affected by genotype, TOS, and leaf surface (all *p* < 0.001) (**Table 5**). *Post-hoc* tests confirmed that adaxial SD exceeded abaxial SD at both TOS (*p* < 0.001) and that SD was higher at TOS 2 than TOS 1 for both surfaces (*p* < 0.001). The lowest SD occurred on the abaxial surface at TOS 1, significantly lower than all other groups (*p* < 0.001) (**Figure 5C**). On average, SD increased from 35.4 to 40.2 mm^−2^ on the abaxial surface and from 46.9 to 50.4 mm^−2^ on the adaxial surface between TOS 1 and TOS 2, representing a 13.5% and 7.3% increase, respectively (**Supplementary Table 6**). Significant TOS × variety (*p* < 0.001), TOS × surface (*p* < 0.05), and variety × surface (*p* < 0.05) interactions indicate that SD variation is both genotype- and surface-dependent (**Figure 7**). A moderate negative correlation was observed between SD and SA (*R*^2^ = 0.312).

**Figure 8:**
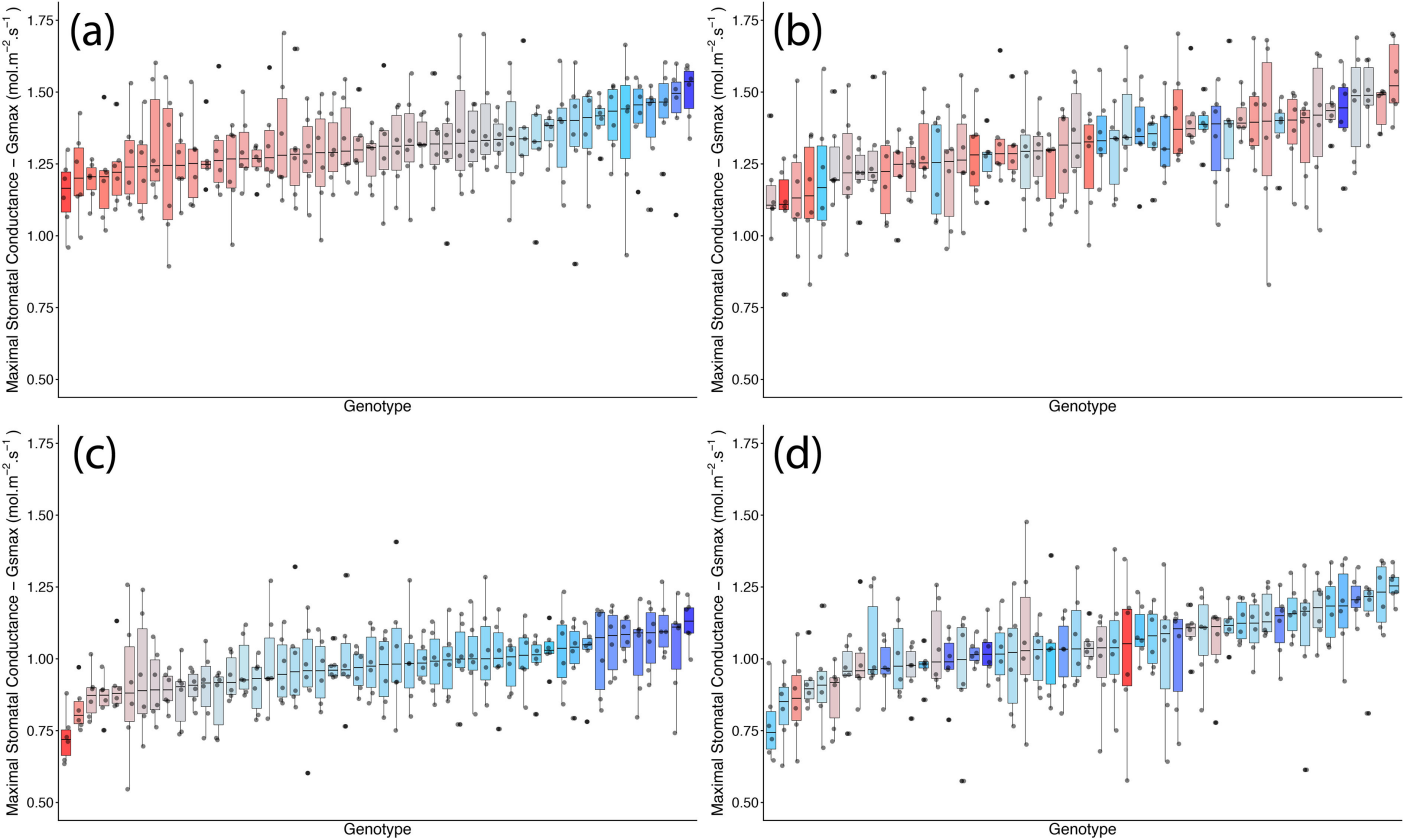
Genotypic distribution of maximum anatomical stomatal conductance (*g_smax_*) across 50 wheat genotypes at two sowing times in season 2. **(A)** Adaxial surface TOS 1; **(B)** adaxial surface TOS 2; **(C)** abaxial surface TOS 1; and **(D)** abaxial surface TOS 2. Genotypes are ranked by median *g_smax_*. Colour assigned to each genotype based on TOS 1 *g_smax_*. Thick horizontal lines within boxes indicate the median and boxes indicate the upper (75%) and lower (25%) quartiles. Whiskers indicate the ranges of the minimum and maximum values. Points indicate individual measurements.Tables.

*g*_smax_ varied significantly amongst genotypes, TOS, and leaf surfaces (all *p* < 0.001) (**Table 5**; **Figure 8**). *Post-hoc* tests showed adaxial *g*_smax_ was consistently higher than abaxial at both TOS (*p* < 0.001). While adaxial *g*_smax_ remained unchanged between TOS, abaxial *g*_smax_ increased by 7.1% at TOS 2 compared with TOS 1 (*p* < 0.001) (**Figure 5D**). On average, abaxial *g*_smax_ rose from 0.97 to 1.04 mol m^−2^ s^−1^, whereas adaxial *g*_smax_ was stable at 1.32 mol m^−2^ s^−1^ across TOS. Significant TOS × genotype (*p* < 0.05), TOS × surface (*p* < 0.001), and genotype × surface (*p* < 0.001) interactions indicate that TOS effects on *g*_smax_ were genotype- and surface-dependent, with leaf surface modulating genotypic responses.

#### Integrated stomatal conductance and anatomy

Significant effects of leaf surface and genotype on *g*_se_ were detected (both *p* < 0.001) (**Table 5**). *Post-hoc* tests confirmed that adaxial *g*_se_ was consistently higher than abaxial across both TOS (*p* < 0.001). No overall decline in *g*_se_ was observed under heat stress (**Figure 5E**). On average, *g*_se_ decreased slightly on the abaxial surface from 0.18 at TOS 1 to 0.14 at TOS 2, whilst adaxial *g*_se_ increased from 0.26 to 0.29 across TOS. Significant TOS × genotype (*p* < 0.001) and TOS × surface (*p* < 0.05) interactions indicate that TOS effects on *g*_se_ were modulated by both genotype and leaf surface.

#### Yield parameters

##### Grain yield

Later sowing significantly reduced yield (*p* < 0.001). Mean grain yield declined from 5.88 t ha^−1^ at TOS 1 to 3.48 t ha^−1^ at TOS 2, representing a 40.8% reduction (**Supplementary Table 6**). Yield also varied significantly amongst the 50 genotypes (*p* < 0.001) (**Table 5**), and a significant TOS × genotype interaction (*p* < 0.001) indicates that the impact of TOS was genotype-dependent. No significant relationships were detected between yield and stomatal traits in S2.

##### Thousand kernel weight

TKW was significantly lower at TOS 2 than at TOS 1 (*p* < 0.001). There was also significant variation in TKW across the 50 genotypes (*p* < 0.001) (**Table 5**). Additionally, the TOS × genotype interaction was significant (*p* < 0.001), suggesting that the effect of TOS on TKW varies dependent on genotype.

##### Screenings percentage

TOS significantly affected screenings percentage (*p* < 0.001), 88.4% higher at TOS 2 than at TOS 1 (*p* < 0.001) (**Supplementary Table 6**). There was also significant variation in screenings percentage across the 50 genotypes (*p* < 0.001) (**Table 5**). Additionally, the TOS × genotype interaction was significant (*p* < 0.001), suggesting that the effect of TOS on screenings percentage varies dependent on genotype.

##### Protein, moisture, and test weight

Protein, moisture, and test weight were all significantly affected by TOS (*p* < 0.001) and genotype (*p* < 0.001) (**Table 5**).

#### Genome–phenome analyses

Similar to S1, stomatal anatomical traits (GCL, GCW, SA, and SD) had the highest heritability estimates (0.296 to 0.763). *g_smax_* had an estimated heritability ranging from 0.048 to 0.658. Stomatal conductance traits, *g*_s_ and *g_se_*, had lower heritability estimates, ranging from 0.089 to 0.252, amongst trials that included detectable heritability estimates (**Table 3**). Noticeably, heritability estimates for *g*_s_ and *g_se_* were only detectable at TOS1 for the abaxial surface.

A total of two putative QTLs for GCL were detected, located on chromosomes 6B (TOS 1; adaxial) and 7A (TOS 2; abaxial) (**Table 4**; **Supplementary Table 5**). Notably, the QTL for GCL at 6B lies in close proximity to another QTL for GCL identified in S1, although the two correspond to different leaf surfaces (**Supplementary Tables S5, S8**).

## Discussion

This study reveals the complex interplay between environmental conditions, stomatal anatomy, and physiological responses and characterises their genetic architecture across a diverse wheat panel, comprising 200 genotypes. We provide a novel perspective by integrating anatomical and physiological traits, thus providing robust evidence on how *g_s_*, stomatal anatomy, operating efficiency, and, ultimately, grain yield are influenced by TOS and genotype. The findings underscore the critical role of stomatal traits in mediating plant adaptation to higher temperatures and associated abiotic stress. Identifying putative QTL that are consistent across seasons (6B), or pleiotropic for multiple traits (1A, 2A, 2B, 4B, 5D, and 6B), highlights their value for understanding the genetic architecture underlying stomatal traits relevant to climate-resilient wheat adaptation.

### Stomatal physiology

In S1, *g*_s_ was strongly influenced by TOS and leaf surface, alongside significant TOS × Variety and TOS × Surface interactions. Earlier sowing (TOS 1) conferred higher *g*_s_ values compared to later sowing (TOS 2), leading to a decline of 28.9%, likely reflecting more favourable cooler conditions. This is consistent with previous findings highlighting the sensitivity of *g_s_* to thermal stress, particularly in cereals, where later sowing compresses reproductive phases into hotter conditions, limiting carbon assimilation and translocation potential (Mahdavi et al., 2021; Ramya et al., 2016; Schoppach et al., 2020).

This trend was not observed in S2, with *g_s_* not significantly different between TOS. The divergence in responses between S1 and S2 likely reflects a combination of inter-annual environmental variation (e.g., heat intensity) and genotypic plasticity. In S1, maximum temperatures in TOS 2 rose to an average of 27.2°C compared with an average maximum of 25.1°C at TOS 2 in S2. The less severe heat stress in S2 likely buffered plants against significant declines in *g_s_* at TOS 2. Additionally, the lack of a TOS effect on *g*_s_ in S2 may relate to the inherent stress tolerance of the germplasm used. Previous studies have shown that certain heat-tolerant wheat cultivars can maintain or even increase *g*_s_ under high temperatures as part of their adaptive strategy to sustain photosynthesis under stress (Abdelhakim et al., 2021; Mirosavljević et al., 2021; Ramya et al., 2021). Genotype-specific resilience may have reduced population-level differences between TOS in S2, highlighting the importance of understanding trait plasticity and genotype-by-environment interactions within breeding-relevant frameworks. *g_s_* responses are not only genotype-dependent but also highly context-specific, with environmental factors such as heat intensity, timing, and stress duration influencing *g_s_* responses to stress.

The adaxial leaf surface exhibited consistently higher *g_s_* than the abaxial surface in both years, reinforcing its role as the primary contributor to leaf gas exchange (Pinto et al., 2025; Shahinnia et al., 2016; Wall et al., 2023, 2022). Similarly, the TOS × Surface interaction suggests that genotypes differ in how stomatal behaviour is partitioned between surfaces under environmental stress, with some showing more balanced or adaptive surface-specific responses. Additionally, the effect of TOS on *g_s_* was most substantial on the adaxial surface in both years, highlighting the functional responsiveness of the adaxial surface in driving leaf gas exchange. This aligns with evidence that adaxial stomata—often more responsive, functionally active, and present at higher densities—play a more critical role in regulating photosynthesis and transpiration than their abaxial counterparts, particularly under higher temperatures (Pinto et al., 2025; Wall et al., 2022).

### QTL for stomatal physiology

The significant TOS × Variety interaction observed across both datasets indicates that genotypes differ not only in their baseline levels of *g_s_* but also in their plasticity to adjust *g_s_* in response to higher temperatures. Such genotype-dependent plasticity demonstrates that genetic control of *g_s_* is environment-specific rather than constitutive, reflecting multiple physiological strategies for heat tolerance. QTLs for *g_s_* have previously been identified in wheat under abiotic stress. Wang et al. (2015), for example, reported six QTLs for *g_s_*, including one on chromosome 7B, a region that in our study was associated exclusively with anatomical traits rather than conductance. This suggests that this region likely influences *g_s_* indirectly via stomatal anatomy, with the functional expression of these traits potentially contingent on environmental context or developmental timing. In our study, no QTL candidates were identified for *g_s_*, aligning with the lower heritability of stomatal physiology traits found both here and in previous literature. Additionally, the QTLs reported for *g_s_* by Wang et al. (2015) had low LOD scores (2.5–2.78), indicating weaker likelihood of trait association.

These findings suggest that both intrinsic stomatal capacity and dynamic regulation differ between genotypes and are modulated by environmental context, particularly TOS, with the genetic architecture underpinning stomatal physiological function appearing less stable and more context-dependent than that of stomatal anatomy. Rather than supporting indirect selection on stomatal conductance traits, these results emphasise the importance of characterising genotype−by−environment interactions and physiological plasticity when interpreting *g_s_* responses under heat stress. Integrating understanding of these adaptive strategies and constraints on selection provides critical context for interpreting stomatal behaviour and its limits under increasing climate variability.

### Stomatal anatomy

Anatomical traits such as GCL, SA, and SD were significantly influenced by TOS and genotype in both seasons, with consistent and strong effects of leaf surface, particularly for SD and GCW. The adaxial surface exhibited significantly higher SD than the abaxial surface in both years, reinforcing established patterns in wheat and other grasses, in contrast to the more common abaxial dominance reported for many non-grass species (McAusland et al., 2021; Shahinnia et al., 2016; Wall et al., 2023, 2022). Significant genetic determination of SD and surface-specific stomatal distribution suggests functional specialisation, with the adaxial surface contributing more prominently to gas exchange under favourable conditions (Samantara et al., 2025).

TOS 1 was consistently associated with larger guard cells and a greater SA, yet by contrast, later sowing (TOS 2) resulted in smaller stomata with reduced SA, found at higher densities. These anatomical shifts, consistent with prior literature, likely represent adaptive developmental responses to increased heat and evaporative demand later in the season (Pinto et al., 2025). The strongest reductions in SA occurred on the adaxial surface, suggesting that it is also the most plastic and environmentally responsive (Hõrak, 2025; Wall et al., 2022).

A negative correlation between SA and SD was observed across both seasons, consistent with a well-established size–density trade-off (Drake et al., 2013; Haworth et al., 2023; Lawson and Blatt, 2014; Pinto et al., 2025). Smaller stomata at higher densities are often associated with faster stomatal kinetics, improving responsiveness to environmental fluctuations, thus enabling plants to optimise the balance between carbon uptake and water loss on short timescales (Drake et al., 2013; McAusland et al., 2016). This can be especially valuable under later sowing conditions where high VPDs and heat spikes create a dynamic stress landscape as a dense arrangement of small stomata may allow tighter and faster control over transpirational water loss without sacrificing photosynthetic capacity. Such an approach aligns with efforts to enhance both intrinsic WUE and the speed and precision of gas exchange regulation, a dual pathway aimed at improving resilience in climate-challenged cropping systems. The elevated adaxial SD observed in our study likely contributes to the higher *g_s_* and *g_smax_* values seen on this surface across both seasons. This anatomical configuration of smaller stomata at greater density may underpin the adaxial surface’s superior capacity for both baseline gas exchange and for dynamic and rapid adjustment under stress (Faralli et al., 2019; McAusland et al., 2021; Pflüger, 2025). Under stressful conditions, the ability to rapidly close stomata can reduce water loss whilst equally rapid reopening can ensure photosynthetic recovery once conditions improve.

The benefits of high SD are not universal amongst stress types and, in some cases, lower SD has been associated with enhanced WUE and drought tolerance whilst negative relationships between SD and *g_smax_* with grain yield have also been reported in wheat, likely reflecting greater total water loss through more numerous adaxial stomata (Dunn et al., 2019; Li et al., 2023, 2017; Samantara et al., 2025). These varying trends where contrasting traits potentially confer resilience in different scenarios suggest that the optimal anatomical ideotype is likely context-dependent and stress-type specific, requiring functional integration with physiological control mechanisms including efficient stomatal kinetics and regulation to fully realise its benefits and confer resilience in different environments.

A higher adaxial *g_smax_* aligns with prior literature, affirming the adaxial surface as the dominant driver of leaf gas exchange (Samantara et al., 2025). That abaxial *g_smax_* increased under stress at TOS 2 is consistent with Pinto et al. (2025). This suggests an adaptive anatomical shift whereby the abaxial surface, typically contributing less to gas exchange, increases its capacity when adaxial function is constrained by heat stress, potentially enhancing transpirational cooling or buffering against reductions in total foliar carbon assimilation (Wall et al., 2022).

The positive correlation between *g_smax_* and *g_sop_* confirms that anatomical potential influences, but does not fully determine, functional gas exchange outcomes (McAusland et al., 2021). *g_sop_* reflects a dynamic interplay where anatomical capacity sets the upper limit, whilst short-term environmental conditions and stomatal regulation determine its actual expression. This underscores the importance of integrating both anatomical and physiological traits in breeding frameworks. SA, SD, and *g_smax_* should be considered alongside dynamic stomatal control to develop wheat varieties that can efficiently balance carbon assimilation and water use under heat stress. That being said, phenotyping dynamic stomatal responses and kinetics *in situ* under field conditions at scale remain a significant challenge, currently limiting the incorporation of these dynamic stomatal traits into breeding pipelines.

### QTL for stomatal anatomy

QTLs for stomatal anatomical traits including stomatal density and size have been previously mapped in wheat, some with pleiotropic loci for yield. Of the 62 putative QTLs we found across five stomatal traits, 21 overlapped with the same chromosomal regions for the same traits previously reported by Ahmed et al. (2021); Liu et al. (2025); Shahinnia et al. (2016), and Wang et al. (2016), supporting both the validity and novelty of our findings (**Supplementary Table 7**). However, whilst overlapping chromosomal regions were identified, it is important to note that due to different genotyping platforms, the specific genomic loci of the markers cannot be mapped across different studies; thus, it cannot be determined whether the 21 overlapping regions related to the same precise QTL. Furthermore, we found two QTLs for SD on chromosome 7A, one of which was associated with a relatively high LOD score (LOD = 6.15), along with one QTL on 7A for GCL. Shahinnia et al. (2016); Ahmed et al. (2021), and Liu et al. (2025) collectively reported 10 QTLs for SD and 3 QTLs for GCL on chromosome 7A, reinforcing the likelihood that this locus has essential genes involved in the regulation of stomatal anatomical traits. Furthermore, QTLs for SD on chromosomes 1A, 2A, 2B, 3A, 4B, 5A, 6B, 7A, and 7B in our study also showed overlap with those previously identified by Liu et al. (2025); Ahmed et al. (2021), and Shahinnia et al. (2016). These loci therefore warrant detailed fine-mapping studies to further validate these QTLs.

Co-located markers at QTL detected across multiple seasons, or sowing times, are strong candidates for marker-assisted selection due to their robustness to environmental variation. Chromosome 1A, for example, contained a QTL for GCW with a closely linked marker position across both sowing times, highlighting potential environmental stability at this locus (**Figure 4**). Beyond stability across sowing times, co-localised QTLs were observed where different traits mapped to the same region with co-localised QTLs for different traits, suggesting shared developmental or regulatory pathways (**Supplementary Table 8**). Of these, chromosome 6B had the equal highest number of QTL identified across four stomatal anatomical traits and included several closely positioned QTL candidates for GCL across seasons and sowing times, suggesting a genomic region with potentially environmentally robust control of stomatal anatomy. Other closely located, co-localised QTLs exhibiting pleiotropic effects for multiple traits include regions in chromosomes 1A (GCW, SA, and SD), 2A (GCL and SA), 2B (GCL and SD), 4B (GCW and SD), 5D (GCW, SA and SD), and 6B (GCW and SA) (**Supplementary Tables S4, S5, S8**). While not all co-located, it is also noteworthy that chromosome 2A contains QTLs across all five stomatal anatomical traits, whilst 1A spans four traits, demonstrating the value of future more detailed mapping at these loci. Additionally, the higher number of QTLs identified for the adaxial surface (36) compared with the abaxial surface (26) reinforces the surface-specific nature of stomatal regulation and the dominant role that the adaxial surface plays in regulating leaf gas exchange. Together, these findings suggest that certain genomic regions exert consistent coordinated control over stomatal anatomy and function, either through pleiotropic genes, or tightly linked loci influencing multiple stomatal traits, whilst also highlighting adaxial dominance. Future directions regarding these candidate QTLs and their translation into breeding targets are discussed later.

### Integrated stomatal conductance and anatomy traits and QTL

*g_se_*, a measure of how closely *g_sop_* operates to its *g_smax_*, was significantly influenced by TOS in S1, by genotype in S2, and by leaf surface in both years, highlighting that *g_se_* is shaped by the intrinsic physiological capacity of genotypes and their plasticity in stomatal behaviour under environmental variation. Higher *g_se_* at TOS 1 across both leaf surfaces in S1 suggests that earlier-sown plants operate closer to their anatomical gas exchange potential under cooler, less stressful conditions. In contrast, the 27.5% decline in *g_se_* at later sowing in S1 points to heat stress impairing stomatal function, resulting in suboptimal gas exchange despite maintained anatomical capacity. However, no such difference between TOS was detected in S2, suggesting that the effect of TOS, and thus heat stress, on *g_se_* may not be uniform across seasons. Such divergence could reflect differences in environmental conditions as previously discussed, genotype performance, or their interaction and highlights the complexity of predicting physiological responses to heat. The patterns we observed, particularly in S1, highlight a decoupling of physiological function (*g_sop_*) from structural potential (*g_smax_*) under stress, aligning with prior work by McAusland et al. (2016) and Ochoa et al. (2024), showing that stomata may exhibit delayed or incomplete opening, even when anatomical capacity remains unchanged, leading to inefficient photosynthetic regulation.

Notably, significant genotype-level variation in *g_se_* in S2 and its interaction with TOS reveals that some varieties possess more effective stomatal regulatory mechanisms, allowing them to maintain efficient gas exchange even under stress. However, the absence of detectable genotypic variation for *g_se_* in S1 may have been due to stronger environmental regulation of stomatal function relative to anatomical capacity. This plasticity in *g_se_* represents a key physiological component of heat resilience and is emerging as a critical indicator of how efficiently genotypes regulate gas exchange under stress. Consistent with this interpretation, *g_se_* exhibited low to moderate heritability, with higher estimates confined to the abaxial surface. Unlike anatomical traits, which showed recurring or co-located QTL, the lack of any QTL candidates for *g_se_* indicates context−dependent genetic influences rather than robust, repeatable loci. The genetic architecture underpinning *g_se_* appears more sensitive to environmental conditions, highlighting the transient nature of genetic signals underlying dynamic stomatal regulation with limited reliability for selection purposes.

Given its environmental sensitivity, moderate heritability, and lack of direct association with yield or genetic markers in this study, these findings position *g_se_* as a contextual trait for characterising stomatal regulation under stress rather than as a selection target with increases in *g_smax_* alongside declines in *g_se_*, highlighting that selection for genotypes with high *g_smax_* alone is inadequate (Franks et al., 2009).

### Implications for grain yield

Grain yield was significantly higher under timely sown conditions compared to delayed sowing in both seasons, and genotypic variation in yield was evident. Importantly, the significant TOS × Genotype interaction indicates that TOS affects yield differently across genotypes. This suggests that the optimal genotype for yield is not universal but rather context-dependent. The ideal combination of stomatal anatomical traits to maximise yield remains unresolved, with some studies linking higher SD and greater *g_smax_* to improved yield and performance, whilst others report negative relationships between SD or *g_smax_* and yield (McAusland et al., 2021; Samantara et al., 2025). Additionally, other studies have found no correlations between SD and yield, thus warranting further investigation (Liao et al., 2005; Shahinnia et al., 2016).

In this study, *g_s_* and *g_se_* showed no consistent association with yield, exhibited low heritability, and associated with no QTLs, indicating limited potential for indirect selection on these traits to drive yield gains. Rather than functioning as reliable predictors of yield, conductance−based traits appear to reflect environmentally responsive physiological behaviour. These results highlight the importance of considering stomatal traits within an integrated, environment−specific framework, where favourable stomatal anatomical and physiological responses may support adaptation without constituting effective indirect selection criteria for yield improvement.

## Limitations and future directions

We recognise some inherent limitations in our study that we address here and highlight areas of future research. This study was conducted at a single location and focused on one developmental stage. Given that stomatal anatomical and physiological traits are known to exhibit strong plasticity across developmental stages and in response to local environmental drivers (Balla et al., 2019; Buckley, 2019; Lawson and Blatt, 2014; Li et al., 2025), sowing-time effects likely reflect interactions between heat exposure, developmental stage, and stress duration, which may constrain the generalisability of the findings across diverse environments and growth phases. Additionally, all measurements were performed under well-watered conditions, and stomatal behaviour under water-limited systems may diverge substantially, particularly the relationship between stomatal traits and yield (Li et al., 2025; Pantha et al., 2025). Under water stress, sustained reductions in stomatal conductance are likely to impose stomatal limitations to photosynthesis that exceed biochemical limitations across the season when compared with heat stress, thereby strengthening links between stomatal traits and yield (Blum, 2009; Richards et al., 2010). In this study, heat stress was imposed via altered TOS, meaning treatments inherently differed in photoperiod. Given that stomatal conductance and daily stomatal rhythms in wheat are responsive to day length, some observed differences between TOS may reflect indirect photoperiod effects in addition to those of temperature stress (Dodd et al., 2005; Yanovsky and Kay, 2001). It is also important to note that the strength of the second season’s results was reduced by the smaller number of genotypes compared to S1, reflected in fewer detected QTLs. Reduced population size lowers statistical power for detecting loci of small to moderate effect and limits the resolution of QTL confidence intervals, particularly for traits with complex genetic control (Holland, 2007; Xu, 2003). Furthermore, whilst we have inferred that stomatal size and density correspond to stomatal responsiveness, the relationships between anatomical traits and dynamic stomatal kinetics is context-dependent and influenced by numerous factors (Lawson and Vialet‐Chabrand, 2019; McAusland et al., 2016); thus, direct measurements of stomatal kinetics in response to environmental fluctuations remain necessary to validate these assumptions.

Moreover, whilst stomatal anatomy and conductance are central components of gas exchange regulation, their breeding utility is likely to depend on interactions with other traits and environmental drivers, including leaf age, canopy architecture, soil moisture status, VPD, and photosynthetic capacity. Consequently, selection based on stomatal traits alone may be insufficient without considering their coordination with whole-plant and canopy-level processes.

To overcome these limitations, future research should expand phenotyping efforts to multiple environments and developmental stages to capture the full spectrum of stomatal behaviour under variable field conditions. Scaling up stomatal trait assessment will benefit from high−throughput approaches, including UAV−based remote sensing such as hyperspectral and thermal imaging and artificial intelligence (AI)−driven analytics, which can provide scalable and precise measurements of stomatal function (Cheng et al., 2024; El-Hendawy et al., 2019; Sadeh et al., 2025). In particular, deeper investigation into stomatal kinetics and their coordination with photosynthetic efficiency and yield performance will refine trait selection for breeding. Novel targeted breeding and genome editing technologies such as Clustered-Regularly-Interspaced-Short-Palindromic-Repeats (CRISPR)-Cas9 efficiently generate precise modifications within a single generation, showing promise in the development of climate resilient wheat (Abdallah et al., 2025; Dunn et al., 2019). Emerging frameworks such as the leveraging multi-omics approaches combined with cutting-edge AI-modelling to engineer stomatal regulatory networks also present opportunities to develop stomatal ideotype plants, highlighting innovative directions for integrating anatomical and dynamic stomatal traits into breeding pipelines (Chaplin et al., 2025b). With respect to candidate QTLs, future studies could experimentally verify these QTLs and their potential through fine−mapping and, importantly, across multiple seasons, sites, and environmental conditions to identify stable and biologically meaningful QTLs. Alternatively, as stomatal anatomical and physiological traits appear to be linked to numerous QTLs across the genome (**Supplementary Table 4**), breeding programs may benefit from developing an extensive training population and using genomic selection for predicting favourable stomatal traits (Meuwissen et al., 2001).

## Conclusions

This study advances our understanding of how stomatal traits are integral to wheat adaptation to different TOS and, in turn, environmental conditions. By disentangling the effects of *g_s_*, stomatal anatomy, and leaf surface, along with quantifying heritability and mapping QTL, we demonstrate that early sowing supports more optimal stomatal function and that genotypes vary significantly in their ability to maintain efficient conductance under thermal stress.

Our results provide clear field-based evidence of environmentally responsive, context-dependent, and genetically controlled stomatal behaviour. Across five stomatal traits, 62 putative QTLs were identified, 21 of which overlapped with previously reported stomatal QTLs in wheat. Stomatal anatomical traits exhibited higher heritability than conductance traits with all identified QTLs and chromosomal regions being for anatomical traits, particularly adaxially. Chromosome 6B emerges as a promising stable candidate genomic region controlling stomatal anatomical traits, identified at an identical chromosomal region across both seasons and sowing times. 1A, 2A, 3A, 6A, and 7B also emerge as strong candidate chromosomal regions associated with a high number of stomatal anatomical traits, including some with co-localisation. Furthermore, chromosome 7A appears as a promising region controlling SD and GCL, aligned with prior work. In contrast, stomatal conductance and efficiency traits were strongly environmentally modulated and showed no association with genetic markers, nor with yield, placing clear constraints on their utility as direct or indirect selection targets for productivity gains. However, integration of anatomical traits with functional traits (*g_s_* and *g_se_*) may aid with characterisation of trait coordination under stress.

The genetic architecture characterised in this study indicates that stomatal anatomical traits—with higher heritability, greater environmental stability, and recurrent co-located genomic regions—represent a more robust focus for understanding adaptive variation and informing environment−specific breeding frameworks. Our results support hypotheses proposing the adaptive value of small, dense stomata for rapid and responsive gas exchange control under stress, yet the ideal combination of anatomical traits for maximising yield remains unresolved and is likely highly context-specific, and may be linked to other environmental factors, including water availability or evaporative demand. Collectively, this work provides a comprehensive field-based characterisation of the physiological responses and genetic architecture of stomatal traits in wheat, a critical and stand-alone step towards understanding wheat adaptation to heat stress.

## Data Availability

The original contributions presented in the study are publicly available. This data can be found here: Zenodo, doi: 10.5281/zenodo.19706766.

## Supplementary material

Supplementary Figure 1 Schematic representation of procedure for using LI-600 porometer to measure abaxial and adaxial surface stomatal conductance.

Supplementary Figure 2 Representative images of stomatal anatomy collected *in situ* using 200x magnification handheld digital microscope in season 1. (A) adaxial and (B) abaxial raw images collected using microscope. (C) and (D) show image with automatically labelled stomata with ellipses using the deep learning model we trained.

Supplementary Figure 3 Representative images of stomatal anatomy collected *in situ* using 400x magnification handheld digital microscope in season 2. (A) adaxial and (B) abaxial raw images collected using microscope. (C) and (D) show image with automatically labelled stomata with ellipses using the deep learning model we trained.

Supplementary Figure 4 The scree plot of the eigenvalues from the principal component analysis of genotype markers. The first 9 principal components are selected for correction to the population structure.

Supplementary Figure 5 Relationship between stomatal density and stomatal area in season 1. Shaded markers represent irrigated plants and unshaded markers represent rainfed plants. Circular markers represent abaxial leaf surface and triangular represent adaxial leaf surface.

Supplementary Figure 6 LOD score of putative QTLs by trait and chromosome. The scatterplot shows the LOD scores of putatative QTLs identified by chromosome and position (in mega-base pairs), with each colour corresponding to a specific year, TOS and surface.

Supplementary Table 1 Overview of germplasm screened in season 1 GRDC field trials.

Supplementary Table 2 Overview of germplasm screened in season 2 of GRDC field trials, selected from the broader University of Sydney genetics programme. All lines grown in season 2 were grown in season 1.

Supplementary Table 3 Season 1 (2023) data showing average values for key traits at TOS 1 and at TOS 2, and % change from TOS 1 to TOS 2. Values are given for each surface independently and for both surfaces averaged.

Supplementary Table 4 The number of distinct putative QTL candidates across all trials by trait and chromosome. The “.” represents 0. The dark green row indicates a chromosome where QTLs span across all five traits. Lighter green rows indicate chromosomes where QTLs span across four traits.

Supplementary Table 5 List of putative QTL candidates for each trait, organized by trial (indexed by Year and TOS) and surface. The chromosome, position (in base pairs), effect size and LOD score for each corresponding QTL are also provided.

Supplementary Table 6 Season 2 (2024) data showing average values for key traits at TOS 1 and at TOS 2, and % change from TOS 1 to TOS 2. Values are given for each surface independently and for both surfaces averaged.

Supplementary Table 7 The number of QTLs reported in literature by trait and chromosome. The last column shows the number of putative QTLs we found for the same trait and chromosome.

Supplementary Table 8 The number of putative QTLs within a 10 Mbp region by chromosome and trait. A region was segmented by clustering marker position using complete-linkage in hierarchical clustering such that no marker in a region would have distance of more than 10Mbp. The 6 bolded rows indicate a region that may contain pleiotropic QTLs for stomatal traits.
