# Supplementary material for: QTLs for heat-induced stomatal anatomy underpin gas exchange variation in field-grown wheat
Source: Front Plant Sci. 2026 May 18;17:1769384. doi: 10.3389/fpls.2026.1769384 (PMC13223016; doi:10.3389/fpls.2026.1769384)
Supplement: Supplementary Figure 1 — Schematic representation of procedure for using LI-600 porometer to measure abaxial and adaxial surface stomatal conductance. [file DataSheet1.pdf]

Supplementary Materials

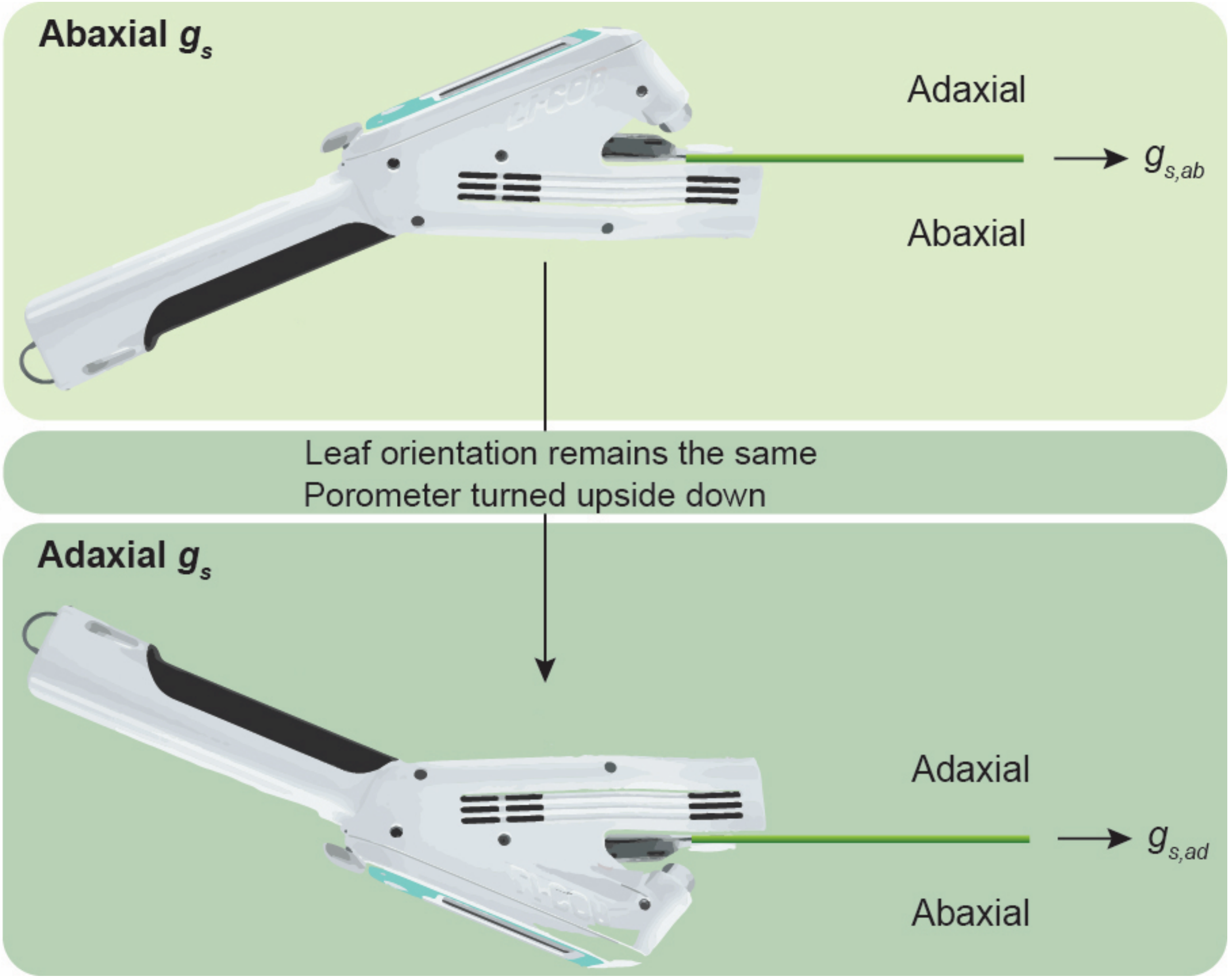

Figure S1: Schematic representation of procedure for using LI-600 porometer to measure abaxial and adaxial surface stomatal conductance.

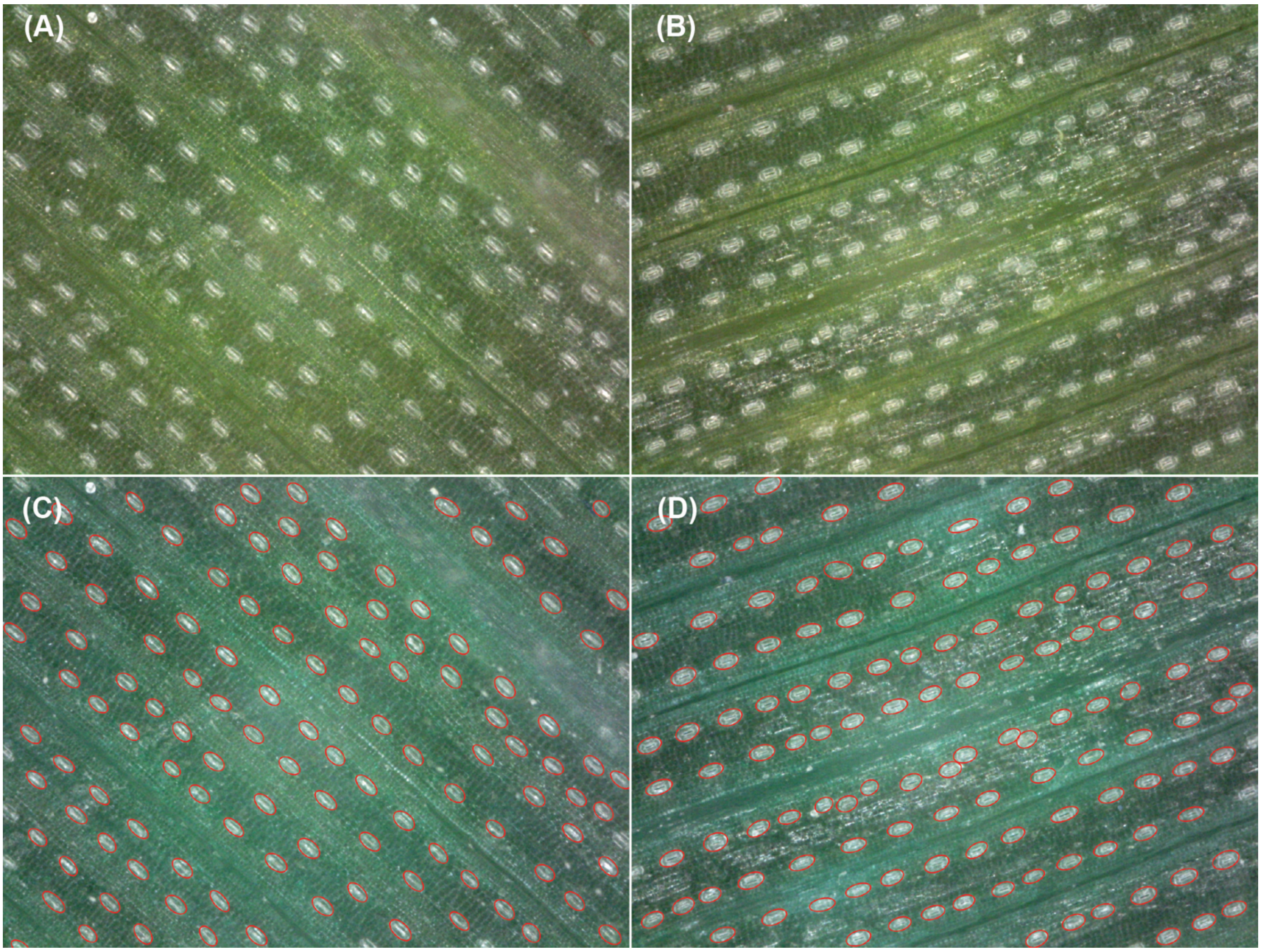

Figure S2: Representative images of stomatal anatomy collected *in situ* using 200x magnification handheld digital microscope in season 1. (A) adaxial and (B) abaxial raw images collected using microscope. (C) and (D) show image with automatically labelled stomata with ellipses using the deep learning model we trained.

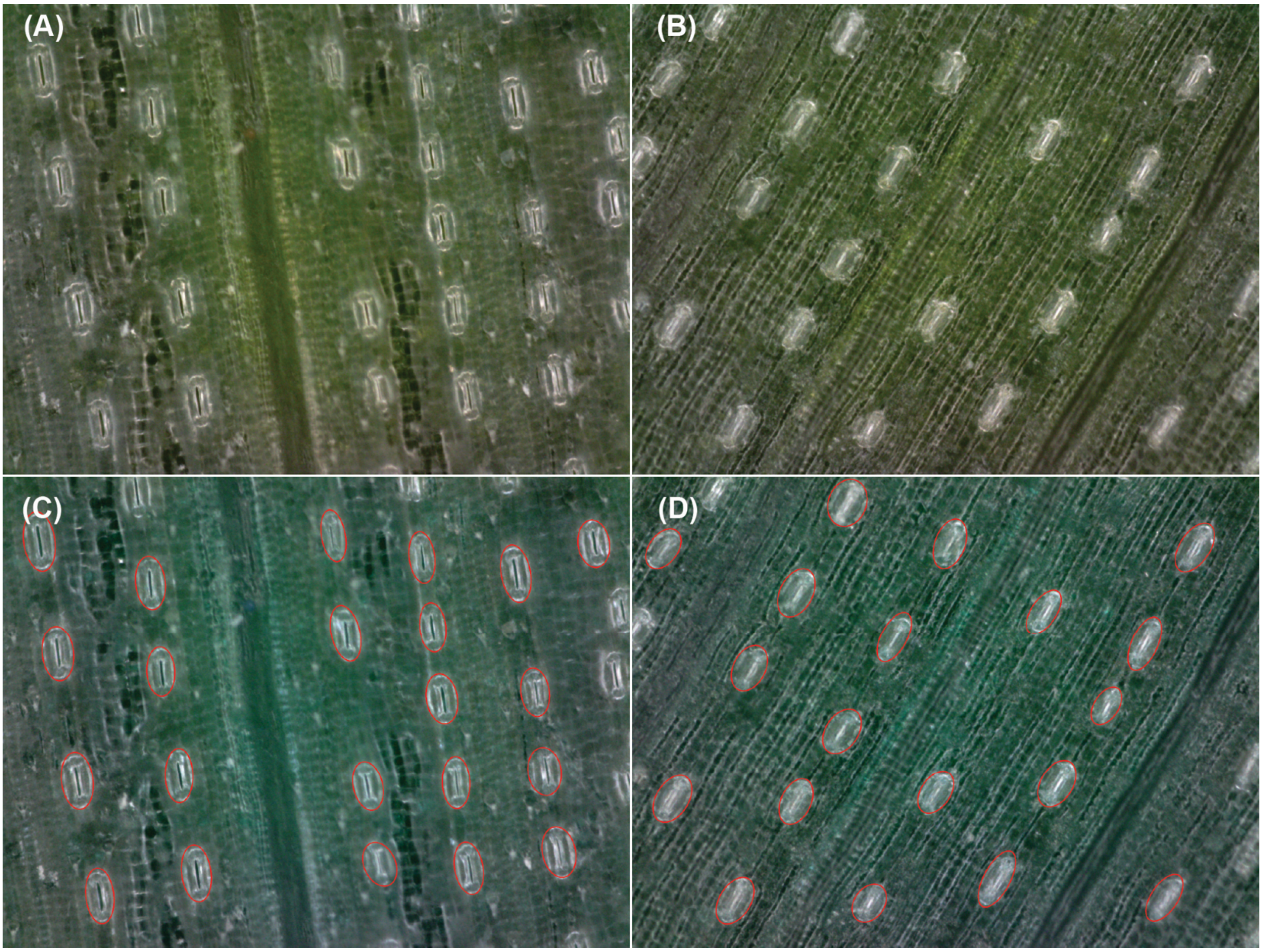

Figure S3: Representative images of stomatal anatomy collected *in situ* using 400x magnification handheld digital microscope in season 2. (A) adaxial and (B) abaxial raw images collected using microscope. (C) and (D) show image with automatically labelled stomata with ellipses using the deep learning model we trained.

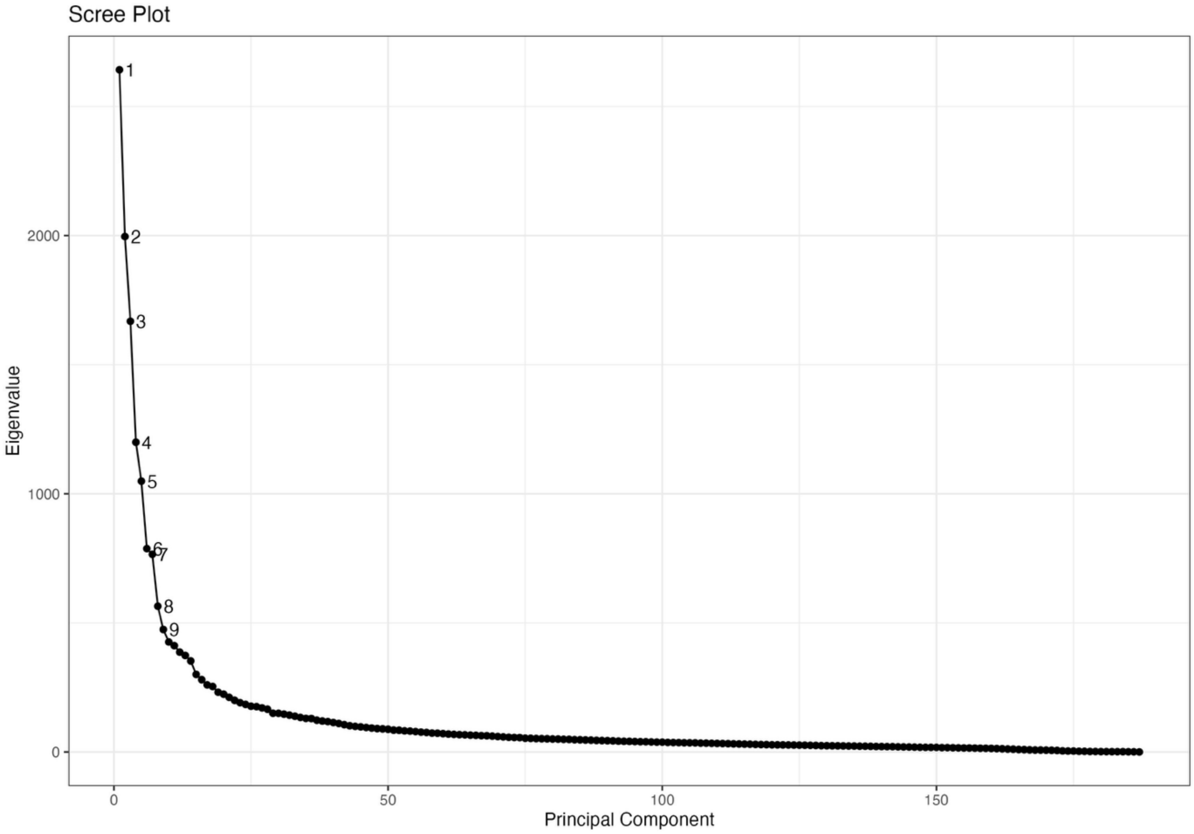

Figure S4: The scree plot of the eigenvalues from the principal component analysis of genotype markers. The first 9 principal components are selected for correction to the population structure.

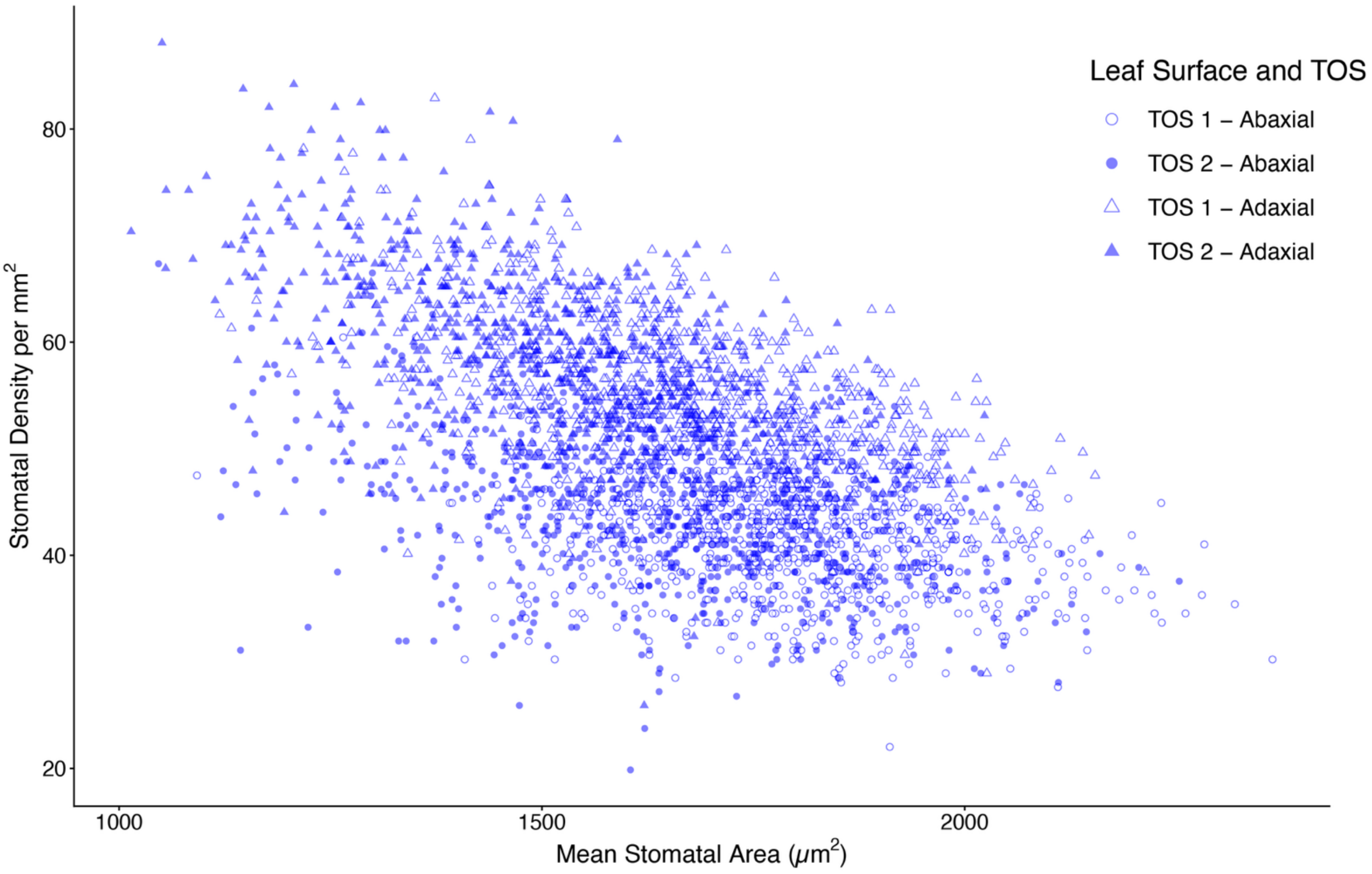

Figure S5: Relationship between stomatal density and stomatal area in season 1. Shaded markers represent irrigated plants and unshaded markers represent rainfed plants. Circular markers represent abaxial leaf surface and triangular represent adaxial leaf surface.

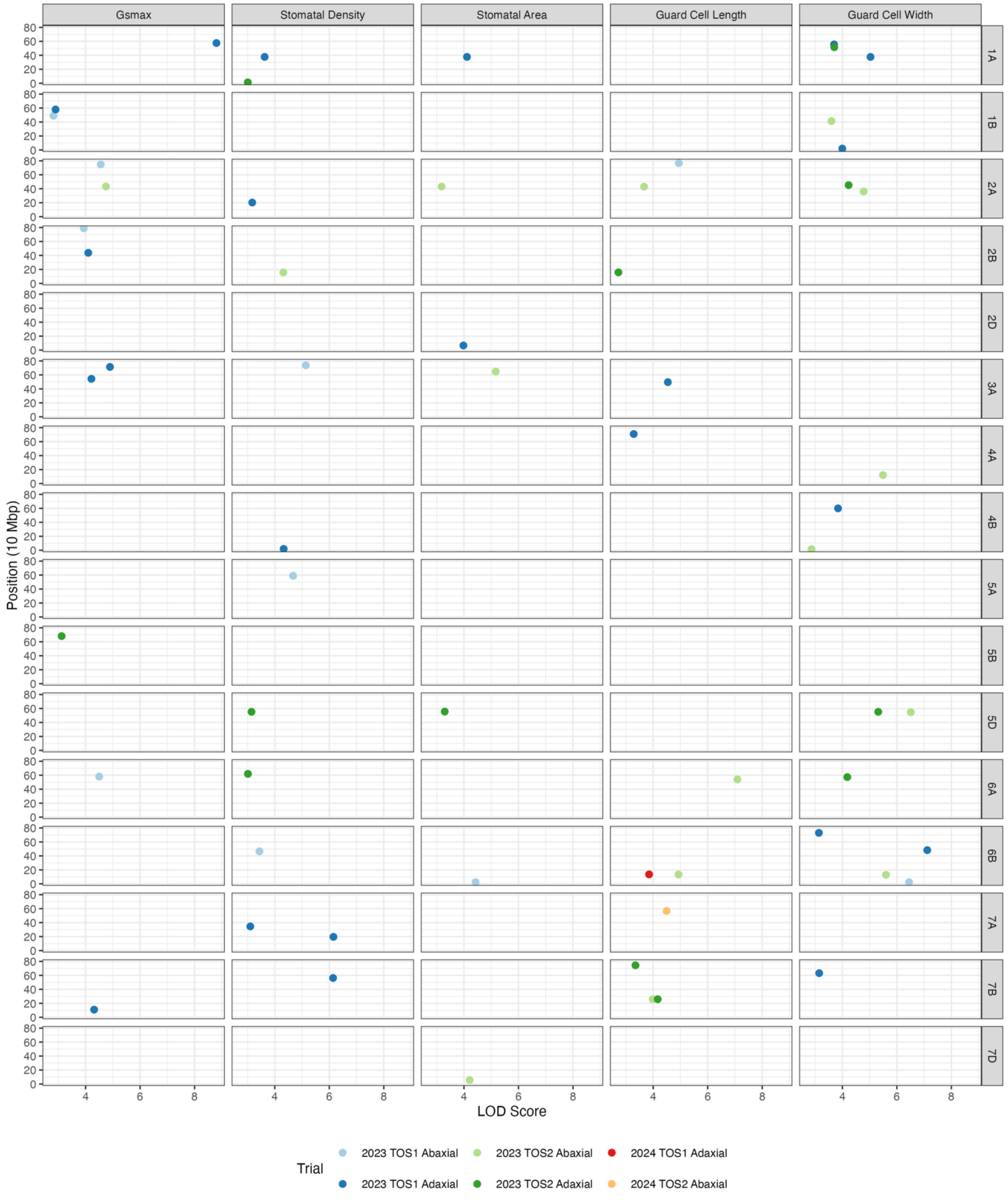

Figure S6: LOD score of putative QTLs by trait and chromosome. The scatterplot shows the LOD scores of putatative QTLs identified by chromosome and position (in mega base pairs), with each colour corresponding to a specific year, TOS and surface.

Table S1: Overview of germplasm screened in season 1 of GRDC field trials.

No.	Designation	No.	Designation
1	PBI09C009-BC-DH80	101	PBI17N014-0C-0N-0N-010N-4N
2	PBI09C008-BC-DH17	102	PBI17N014-0C-0N-0N-010N-5N
3	PBI09C043-BC-0C-20N-99N	103	PBI17N014-0C-0N-0N-010N-6N
4	PBI09C048-BC-0C-6N-99N	104	PBI17N014-0C-0N-0N-010N-8N
5	ACIAR09PBI C04-52C-DH3	105	PBI17N014-0C-0N-0N-010N-16N
6	ACIAR09PBI C04-52C-DH10	106	PBI17N014-0C-0N-0N-010N-19N
7	PBI07C101-DH96	107	PBI17N015-0C-0N-0N-010N-2N
8	PBI07C101-DH147	108	PBI17N015-0C-0N-0N-010N-5N
9	CMSA05Y00954T-040M-040ZTP0Y-040ZTM-040SY-12ZTM-01Y-0B	109	PBI17N015-0C-0N-0N-010N-8N
10	SUNTOP	110	PBI17N015-0C-0N-0N-010N-10N
11	ICW02.00099-11APTS-0AP-0AP-9AP-0AP	111	PBI17N015-0C-0N-0N-010N-19N
12	ICW04.20024-9AP-0AP-0AP-0AP-9AP-0AP	112	PBI17N015-0C-0N-0N-010N-24N
13	CMSS08B00645T-099TOPY-099M-099NJ-099NJ-6WGY-0B	113	PBI17N016-0C-0N-0N-010N-27N
14	CMSS08B00648T-099TOPY-099M-099NJ-2WGY-0B	114	PBI17N018-0C-0N-0N-010N-1N
15	CMSA08Y00613S-050Y-050ZTM-050Y-59BMX-010Y-0B	115	PBI17N019-0C-0N-0N-010N-6N
16	CMSA08M00007T-030(AWW5L7BO1HET)Y-040M-0NJ-4Y-0B	116	PBI17N020-0C-0N-0N-010N-12N
17	SUNLIN	117	PBI17N021-0C-0N-0N-010N-5N
18	CMSS06Y00885T-099TOPM-099Y-099ZTM-099NJ-099NJ-26WGY-0B	118	PBI16C001-0C-0N-0N-010N-5N
19	CMSA06M00008T-024(PINBD1BHET)Y-040ZTM-026(PINBD1BPOS)ZTY-20ZTM-0Y-0B	119	PBI16C007-0C-0N-0N-010N-2N
20	CMSA05M00140S-021(CRE1M19-GWM577BO1)M-028(BO1 POS& CRE1 POS)ZTY-040ZTM-040SY-11ZTM-0Y-0B	120	PBI16C008-0C-0N-0N-010N-7N
21	MACE	121	PBI16C009-0C-0N-0N-010N-14N
22	SCOUT	122	PBI16C009-0C-0N-0N-010N-15N
23	CMSA05Y01186T-040M-040ZTP0Y-040ZTM-040SY-32ZTM-02Y-0B	123	PBI16C009-0C-0N-0N-010N-17N
24	CGSS05B00258T-099TOPY-099M-099Y-099ZTM-12WGY-0B	124	PBI16C010-0C-0N-0N-010N-6N
25	SUNCO	125	PBI16C011-0C-0N-0N-010N-1N
26	BIOINTA 1005	126	PBI16C011-0C-0N-0N-010N-5N

27	BORLAUG 100	127	PBI16C011-0C-0N-0N-010N-10N
28	COOLAH	128	PBI16C013-0C-0N-0N-010N-4N
29	CUTLASS	129	PBI16C013-0C-0N-0N-010N-12N
30	SCEPTER	130	PBI16C015-0C-0N-0N-010N-10N
31	CONDO	131	PBI16C015-0C-0N-0N-010N-13N
32	FLANKER	132	PBI16C016-0C-0N-0N-010N-11N
33	VIKING	133	PBI16C016-0C-0N-0N-010N-14N
34	ICW05.0660-18AP-0AP-0AP-1AP-0AP	134	TUK004-0Q-0N-020N-0N-5N
35	CMSS11B00167S-099M-0SY-11M-0WGY	135	TUK004-0Q-0N-020N-0N-8N
36	PTSS02Y00021S-099B-099Y-030ZTM-040SY-040M-21Y-0M-0SY-0Y-0Y	136	TUK004-0Q-0N-020N-0N-36N
37	PTSS02Y00023S-099B-099Y-099B-099Y-100B-0Y	137	PTSS15Y00010S-099B-099Y-099M-2Y-020Y
38	PTSS08GHB00013S-0SH-099SHB-099SHB-099SHB-32Y-0Y	138	PTSS15Y00023S-099B-099Y-099M-23Y-020Y
39	PTSS08GHB00016S-099SH-099Y-099SHB-099Y-155Y-0Y	139	PTSS15Y00024S-099B-099Y-099M-2Y-020Y
40	SDSS12B00849T-0Y-0B-0B-12Y-0B	140	PTSS15Y00024S-099B-099Y-099M-10Y-020Y
41	SUNCHASER	141	PTSS15Y00024S-099B-099Y-099M-17Y-020Y
42	VIXEN	142	PTSS14Y00062S-0B-099Y-099B-39Y-020Y
43	IMPALA	143	PTSS14Y00071S-0B-099Y-099B-33Y-020Y
44	RELIANT	144	PTSS14Y00328S-0B-099Y-099B-32Y-020Y
45	BECKOM	145	PTSS14Y00329S-0B-099Y-099B-33Y-020Y
46	HELLFIRE	146	PTSS11Y00152S-0SHB-099B-099Y-099B-099Y-17Y-020Y-0B
47	MUSTANG	147	PTSS15Y00053S-099B-099Y-099M-8Y-020Y-0B
48	PBIC15039-0C-9N-010N-2N-0N	148	PTSS15Y00056S-099B-099Y-099M-14Y-020Y-0B
49	PBIC15038-0C-29N-010N-1N-0N	149	PTSS15Y00082S-099B-099Y-099M-16Y-020Y-0B
50	PBIC15034-0C-24N-010N-1N-0N	150	PTSS02B00102T-0TOPY-0B-0Y-0B-11Y-0M-0SY-0B-0Y-4Y-0M
51	PBIC15034-0C-63N-010N-1N-0N	151	PTSS14Y00330S-0B-099Y-099B-40Y-0B
52	PBIC15034-0C-73N-010N-1N-0N	152	PTSS14Y00057S-0B-099Y-099B-10Y-020Y-0B

53	PBIC15030-0C-13N-010N-1N-0N	153	PTSS14Y00013S-0B-099Y-099B-14Y-020Y-0B
54	PBI19N002-0N-5N	154	PTSS15Y00032S-099B-099Y-099M-25Y-020Y
55	PBI19N003-0N-11N	155	PTSS15Y00050S-099B-099Y-099M-1Y-020Y
56	PBI19N007-0N-3N	156	CATAPULT
57	PBI19N007-0N-6N	157	SUNMASTER
58	PBI19N007-0N-8N	159	ROCKSTAR
59	PBI17N001-0N-0N-12N	158	SHERIFF CL PLUS
60	PBI17N002-0N-0N-38N	160	VALIANT CL PLUS
61	PBI17N002-0N-0N-76N	161	HAVOC
62	PBI17N003-0N-0N-32N	162	STEALTH
63	PBI17N004-0N-0N-16N	163	IG-ANU-HeatLine-001
64	PBI17N004-0N-0N-18N	164	IG-ANU-HeatLine-002
65	PBI17N005-0N-0N-29N	165	IG-ANU-HeatLine-008
66	PBI17N005-0N-0N-32N	166	IG-ANU-HeatLine-009
67	PBI17N006-0N-0N-89N	167	IG-ANU-HeatLine-011
68	PBI17N006-0N-0N-96N	168	IG-ANU-HeatLine-023
69	PBI17N007-0N-0N-13N	169	IG-ANU-HeatLine-028
70	PBI17N008-0N-0N-10N	170	IG-ANU-HeatLine-033
71	PBI17N008-0N-0N-113N	171	IG-ANU-HeatLine-034
72	PBI17N009-0N-0N-4N	172	IG-ANU-HeatLine-038
73	PBI17N009-0N-0N-46N	173	IG-ANU-HeatLine-055
74	PBI17N011-0N-0N-20N	174	IG-ANU-HeatLine-056
75	PBI17N011-0N-0N-41N	175	IG-ANU-HeatLine-074
76	PBI17N012-0N-0N-106N	176	IG-ANU-HeatLine-078
77	PBI17N013-0N-0N-3N	177	IG-ANU-HeatLine-080
78	PBI17N013-0N-0N-62N	178	IG-ANU-HeatLine-081
79	PBI17N014-0N-0N-54N	179	IG-ANU-HeatLine-088
80	PBI17N015-0N-0N-58N	180	IG-ANU-HeatLine-097
81	PBI17N015-0N-0N-96N	181	IG-ANU-HeatLine-103
82	PBI17N001-0C-0N-0N-010N-8N	182	IG-ANU-HeatLine-107
83	PBI17N002-0C-0N-0N-010N-16N	183	IG-ANU-HeatLine-010
84	PBI17N004-0C-0N-0N-010N-5N	184	IG-ANU-HeatLine-025
85	PBI17N005-0C-0N-0N-010N-8N	185	IG-ANU-HeatLine-026
86	PBI17N005-0C-0N-0N-010N-10N	186	IG-ANU-HeatLine-035
87	PBI17N005-0C-0N-0N-010N-14N	187	IG-ANU-HeatLine-037
88	PBI17N006-0C-0N-0N-010N-3N	188	IG-ANU-HeatLine-047

89	PBI17N007-0C-0N-0N-010N-8N	189	IG-ANU-HeatLine-051
90	PBI17N007-0C-0N-0N-010N-11N	190	IG-ANU-HeatLine-059
91	PBI17N007-0C-0N-0N-010N-15N	191	IG-ANU-HeatLine-063
92	PBI17N009-0C-0N-0N-010N-10N	192	IG-ANU-HeatLine-067
93	PBI17N010-0C-0N-0N-010N-5N	193	SHAMIEKH-3
94	PBI17N010-0C-0N-0N-010N-6N	194	ALATHEER-4
95	PBI17N010-0C-0N-0N-010N-10N	195	ISR 812.8/CARINYA
96	PBI17N010-0C-0N-0N-010N-13N	196	CMSS08B00648T-099TOPY-099M-099NJ-2WGY-0B
97	PBI17N012-0C-0N-0N-010N-17N	197	PBI16C001-0C-0N-0N-010N-12N
98	PBI17N013-0C-0N-0N-010N-7N	198	CMSA05Y00954T-040M-040ZTP0Y-040ZTM-040SY-12ZTM-01Y-0B
99	PBI17N013-0C-0N-0N-010N-8N	199	PTSS02B00096T-0TOPY-0B-0Y-0B-33Y-0M-0SY-0Y-0Y
100	PBI17N013-0C-0N-0N-010N-9N	200	PTSS02B00094T-0TOPY-0B-0Y-0B-3Y-0ZTB-0SY-0Y-0Y

Table S2: Overview of germplasm screened in season 2 of GRDC field trials, selected from the broader University of Sydney genetics program. All lines grown in season 2 were grown in season 1.

No.	Designation	No.	Designation
1	Borlaug 100	26	PBI17N002-0C-0N-0N-010N-16N
2	Cutlass	27	PBI17N005-0N-0N-32N
3	Hellfire	28	PBI17N006-0N-0N-89N
4	IG-ANU-HeatLine-008	29	PBI17N007-0C-0N-0N-010N-8N
5	IG-ANU-HeatLine-009	30	PBI17N007-0N-0N-13N
6	IG-ANU-HeatLine-010	31	PBI17N009-0N-0N-46N
7	IG-ANU-HeatLine-011	32	PBI17N011-0N-0N-20N
8	IG-ANU-HeatLine-023	33	PBI17N013-0C-0N-0N-010N-8N
9	IG-ANU-HeatLine-037	34	PBI17N014-0C-0N-0N-010N-19N
10	IG-ANU-HeatLine-038	35	PBI17N015-0C-0N-0N-010N-10N
11	IG-ANU-HeatLine-047	36	PBI17N015-0C-0N-0N-010N-8N
12	IG-ANU-HeatLine-051	37	PBI17N015-0N-0N-96N
13	IG-ANU-HeatLine-055	38	PBI17N016-0C-0N-0N-010N-27N
14	IG-ANU-HeatLine-056	39	PBI17N019-0C-0N-0N-010N-6N
15	IG-ANU-HeatLine-059	40	PBI19N007-0N-3N
16	IG-ANU-HeatLine-063	41	PBI19N007-0N-8N
17	IG-ANU-HeatLine-103	42	PBIC15034-0C-24N-010N-1N-0N
18	IG-ANU-HeatLine-107	43	Reliant
19	Mace	44	RockStar
20	Mustang	45	Scepter
21	PBI07C101-DH96	46	Scout
22	PBI16C009-0C-0N-0N-010N-14N	47	Stealth
23	PBI16C013-0C-0N-0N-010N-4N	48	Sunchaser
24	PBI16C016-0C-0N-0N-010N-14N	49	Sunco
25	PBI17N001-0C-0N-0N-010N-8N	50	Suntop

Table S3: Season 1 (2023) data showing average values for key traits at TOS 1 and at TOS 2, and % change from TOS 1 to TOS 2. Values are given for each surface independently and for both surfaces averaged.

Trait & Surface		TOS 1	TOS 2	% Change TOS 1 to TOS 2
**Abaxial**				
g_s	mol.m^{-2}.s^{-1}	0.065	0.055	-14.77
**Stomatal Density**	per mm^{-2}	41.58	44.23	6.37
**Guard cell Length**	µm	64.73	62.19	-3.93
**Guard Cell Width**	µm	39.62	38.44	-2.99
**Stomatal Area**	µm^{-2}	1785.90	1658.94	-7.11
g_{smax}	mol.m^{-2}.s^{-1}	1.13	1.16	2.11
g_{se}	-	0.06	0.05	-15.25
**Adaxial**				
g_s	mol.m^{-2}.s^{-1}	0.186	0.123	-33.89
**Stomatal Density**	per mm^{-2}	54.84	58.52	6.70
**Guard cell Length**	µm	65.86	61.81	-6.14
**Guard Cell Width**	µm	36.99	35.91	-2.91
**Stomatal Area**	µm^{-2}	1672.63	1516.58	-9.33
g_{smax}	mol.m^{-2}.s^{-1}	1.52	1.52	0.06
g_{se}	-	0.12	0.08	-33.33
**Adaxial & Abaxial Average**				
g_s	mol.m^{-2}.s^{-1}	0.125	0.089	-28.93
**Stomatal Density**	per mm^{-2}	48.21	51.37	6.56
**Guard cell Length**	µm	65.30	62.00	-5.05
**Guard Cell Width**	µm	38.31	37.17	-2.95
**Stomatal Area**	µm^{-2}	1729.27	1587.76	-8.18
g_{smax}	mol.m^{-2}.s^{-1}	1.33	1.34	0.93
g_{se}	-	0.09	0.07	-27.53
**Yield**	t.ha^{-1}	4.62	2.79	-39.64

Table S4: The number of distinct putative QTL candidates across all trials by trait and chromosome. The "." represents 0. The dark green row indicates a chromosome where QTLs span across all five traits. Lighter green rows indicate chromosomes where QTLs span across four traits.

Chromosome	GCW	GCL	SA	SD	gsmax	Total QTL
**1A**	3	.	1	2	1	**7**
**1B**	2	.	.	.	2	**4**
**2A**	2	2	1	1	2	**8**
**2B**	.	1	.	1	2	**4**
**2D**	.	.	1	.	.	**1**
**3A**	.	1	1	1	2	**5**
**4A**	1	1	.	.	.	**2**
**4B**	2	.	.	1	.	**3**
**5A**	.	.	.	1	.	**1**
**5B**	.	.	.	.	1	**1**
**5D**	2	.	1	1	.	**4**
**6A**	1	1	.	1	1	**4**
**6B**	4	2	1	1	.	**8**
**7A**	.	1	.	2	.	**3**
**7B**	1	3	.	1	1	**6**
**7D**	.	.	1	.	.	**1**
**Total**	**18**	**12**	**7**	**13**	**12**	**62**

Table S5: List of putative QTL candidates for each trait, organized by trial (indexed by Year and TOS) and surface. The chromosome, position (in base pairs), effect size and LOD score for each corresponding QTL are also provided.

Trait	Surface	TOS	Year	Chromosome	Position (bp)	Size	LOD
Guard Cell Length	Abaxial	TOS 1	2023	2A	766,921,434	1.04	4.94
Guard Cell Length	Adaxial	TOS 1	2023	3A	496,449,626	-1.74	4.54
Guard Cell Length	Adaxial	TOS 1	2023	4A	708,765,853	1.02	3.29
Guard Cell Length	Abaxial	TOS 2	2023	2A	430,482,958	1.71	3.67
Guard Cell Length	Abaxial	TOS 2	2023	6A	540,257,365	1.36	7.09
Guard Cell Length	Abaxial	TOS 2	2023	6B	133,455,950	1.32	4.93
Guard Cell Length	Abaxial	TOS 2	2023	7B	258,507,945	-2.18	3.98
Guard Cell Length	Adaxial	TOS 2	2023	2B	157,851,987	0.88	2.72
Guard Cell Length	Adaxial	TOS 2	2023	7B	258,508,293	-2.46	4.17
Guard Cell Length	Adaxial	TOS 2	2023	7B	744,070,052	-1.27	3.35
Guard Cell Length	Adaxial	TOS 1	2024	6B	135,512,355	-1.96	3.85

Guard Cell Length	Abaxial	TOS2	2024	7A	567,092,815	4.20	4.49
Guard Cell Width	Abaxial	TOS1	2023	6B	23,602,849	-0.87	6.45
Guard Cell Width	Adaxial	TOS1	2023	1A	376,842,796	1.95	5.03
Guard Cell Width	Adaxial	TOS1	2023	1A	556,015,897	-0.89	3.70
Guard Cell Width	Adaxial	TOS1	2023	1B	19,858,139	0.56	4.00
Guard Cell Width	Adaxial	TOS1	2023	4B	600,005,003	0.57	3.84
Guard Cell Width	Adaxial	TOS1	2023	6B	482,801,231	-0.95	7.12
Guard Cell Width	Adaxial	TOS1	2023	6B	729,856,201	0.61	3.14
Guard Cell Width	Adaxial	TOS1	2023	7B	632,136,970	0.50	3.15
Guard Cell Width	Abaxial	TOS2	2023	1B	413,270,635	-0.50	3.60
Guard Cell Width	Abaxial	TOS2	2023	2A	360,890,403	0.84	4.78
Guard Cell Width	Abaxial	TOS2	2023	4A	121,156,643	1.21	5.49
Guard Cell Width	Abaxial	TOS2	2023	4B	15,857,336	0.52	2.87

Guard Cell Width	Abaxial	TOS 2	202 3	5D	547,047,05 3	0.81	6.5 1
Guard Cell Width	Abaxial	TOS 2	202 3	6B	129,276,76 0	0.99	5.6 0
Guard Cell Width	Adaxial	TOS 2	202 3	1A	515,621,80 6	-0.52	3.7 0
Guard Cell Width	Adaxial	TOS 2	202 3	2A	451,941,37 6	1.05	4.2 3
Guard Cell Width	Adaxial	TOS 2	202 3	5D	552,286,73 8	0.60	5.3 2
Guard Cell Width	Adaxial	TOS 2	202 3	6A	573,107,47 7	-0.56	4.1 8
Stomata l Area	Abaxial	TOS 1	202 3	6B	23,128,659	-51.25	4.4 3
Stomata l Area	Adaxial	TOS 1	202 3	1A	376,842,79 6	162.8 8	4.1 1
Stomata l Area	Adaxial	TOS 1	202 3	2D	66,178,968	52.17	3.9 8
Stomata l Area	Abaxial	TOS 2	202 3	2A	430,482,95 8	84.81	3.1 8
Stomata l Area	Abaxial	TOS 2	202 3	3A	648,742,42 6	-87.71	5.1 6
Stomata l Area	Abaxial	TOS 2	202 3	7D	56,579,964	62.85	4.2 1
Stomata l Area	Adaxial	TOS 2	202 3	5D	555,937,21 4	51.35	3.2 9
Stomata l Density	Abaxial	TOS 1	202 3	3A	737,463,56 3	-0.24	5.1 4
Stomata l Density	Abaxial	TOS 1	202 3	5A	589,269,77 4	-0.12	4.6 7
Stomata l Density	Abaxial	TOS 1	202 3	6B	465,378,91 6	-0.11	3.4 4

Stomatal Density	Adaxial	TOS1	2023	1A	377,335,032	0.28	3.63
Stomatal Density	Adaxial	TOS1	2023	2A	202,235,244	-0.17	3.17
Stomatal Density	Adaxial	TOS1	2023	4B	19,562,029	0.12	4.33
Stomatal Density	Adaxial	TOS1	2023	7A	195,925,642	-0.22	6.15
Stomatal Density	Adaxial	TOS1	2023	7A	345,399,193	-0.19	3.10
Stomatal Density	Adaxial	TOS1	2023	7B	562,877,494	-0.19	6.14
Stomatal Density	Abaxial	TOS2	2023	2B	157,852,195	0.16	4.31
Stomatal Density	Adaxial	TOS2	2023	1A	12,360,105	0.20	3.01
Stomatal Density	Adaxial	TOS2	2023	5D	552,685,132	-0.16	3.15
Stomatal Density	Adaxial	TOS2	2023	6A	619,599,375	-0.36	3.02
Gsmax	Abaxial	TOS1	2023	1B	489,763,998	-0.05	2.82
Gsmax	Abaxial	TOS1	2023	2A	748,305,390	0.05	4.56
Gsmax	Abaxial	TOS1	2023	2B	789,702,543	-0.07	3.93
Gsmax	Abaxial	TOS1	2023	6A	581,018,698	0.07	4.50
Gsmax	Adaxial	TOS1	2023	1A	575,536,483	0.07	8.80
Gsmax	Adaxial	TOS1	2023	1B	579,462,182	0.03	2.90
Gsmax	Adaxial	TOS1	2023	2B	437,736,153	-0.07	4.10
Gsmax	Adaxial	TOS1	2023	3A	543,868,879	-0.10	4.21

Gsmax	Adaxial	TOS1	2023	3A	714,228,637	-0.04	4.90
Gsmax	Adaxial	TOS1	2023	7B	109,101,574	0.08	4.31
Gsmax	Abaxial	TOS2	2023	2A	431,666,584	0.07	4.75
Gsmax	Adaxial	TOS2	2023	5B	681,423,212	0.05	3.12

Table S6: Season 2 (2024) data showing average values for key traits at TOS 1 and at TOS 2, and % change from TOS 1 to TOS 2. Values are given for each surface independently and for both surfaces averaged.

Trait & Surface		TOS 1	TOS 2	% Change TOS 1 to TOS 2
**Abaxial**				
g_s	mol.m^{-2}.s^{-1}	0.171	0.137	-19.89
**Stomatal Density**	per mm^{-2}	35.38	40.16	13.52
**Guard cell Length**	µm	65.31	61.72	-5.50
**Guard Cell Width**	µm	38.42	38.58	0.41
**Stomatal Area**	µm^{-2}	1947.47	1855.96	-4.70
g_{smax}	mol.m^{-2}.s^{-1}	0.97	1.04	7.14
g_{se}	-	0.18	0.14	-23.64
**Adaxial**				
g_s	mol.m^{-2}.s^{-1}	0.347	0.374	7.90
**Stomatal Density**	per mm^{-2}	46.94	50.36	7.29
**Guard cell Length**	µm	66.87	62.30	-6.83
**Guard Cell Width**	µm	35.88	36.02	0.41
**Stomatal Area**	µm^{-2}	1844.24	1730.59	-6.16
g_{smax}	mol.m^{-2}.s^{-1}	1.32	1.32	-0.28
g_{se}	-	0.26	0.29	9.45
**Adaxial & Abaxial Average**				
g_s	mol.m^{-2}.s^{-1}	0.259	0.256	-1.27
**Stomatal Density**	per mm^{-2}	41.16	45.26	9.97
**Guard cell Length**	µm	66.09	62.01	-6.17
**Guard Cell Width**	µm	37.15	37.30	0.41
**Stomatal Area**	µm^{-2}	1895.86	1793.27	-5.41
g_{smax}	mol.m^{-2}.s^{-1}	1.15	1.18	2.87
g_{se}	-	0.22	0.21	-3.87
**Yield**	t.ha^{-1}	5.88	3.48	-40.75
**Thousand Kernel Weight**	g	41.16	34.96	-15.06
**Screenings**	%	3.09	5.82	88.43
**Moisture**	%	10.48	9.86	-5.87
**Protein**	%	12.05	12.84	6.54
**Test Weight**	kg.hL^{-1}	83.82	79.77	-4.83

Table S7: The number of QTLs reported in literature by trait and chromosome. The last column shows the number of putative QTLs we found for the same trait and chromosome.

Trait	Chromosome	# Reported QTLs	Source	# Putative QTLs found in our study
SA	1A	3	Ahmed et al. 2021	1
SA	1B	7	Shahinnia et al. 2016, Liu et al. 2025, Ahmed et al. 2021	0
SA	2A	3	Ahmed et al. 2021	1
SA	2B	3	Liu et al. 2025, Ahmed et al. 2021	0
SA	2D	2	Ahmed et al. 2021	1
SA	3B	3	Ahmed et al. 2021	0
SA	3D	2	Ahmed et al. 2021	0
SA	4A	1	Shahinnia et al. 2016	0
SA	4B	9	Shahinnia et al. 2016, Liu et al. 2025, Ahmed et al. 2021	0
SA	5A	4	Shahinnia et al. 2016, Liu et al. 2025, Ahmed et al. 2021	0
SA	5B	19	Shahinnia et al. 2016, Liu et al. 2025, Ahmed et al. 2021	0
SA	5D	3	Shahinnia et al. 2016, Liu et al. 2025	1
SA	6B	5	Ahmed et al. 2021	1
SA	7A	7	Ahmed et al. 2021	0
SD	1A	4	Liu et al. 2025, Ahmed et al. 2021	2
SD	1B	2	Ahmed et al. 2021	0
SD	1D	2	Ahmed et al. 2021	0
SD	2A	6	Ahmed et al. 2021	1
SD	2B	1	Ahmed et al. 2021	1

SD	2D	1	Ahmed et al. 2021	0
SD	3A	7	Shahinnia et al. 2016, Liu et al. 2025, Ahmed et al. 2021	1
SD	3B	8	Ahmed et al. 2021	0
SD	4A	6	Shahinnia et al. 2016, Ahmed et al. 2021	0
SD	4B	8	Shahinnia et al. 2016, Liu et al. 2025, Ahmed et al. 2021	1
SD	5A	11	Shahinnia et al. 2016, Liu et al. 2025, Ahmed et al. 2021	1
SD	5B	3	Shahinnia et al. 2016, Liu et al. 2025, Ahmed et al. 2021	0
SD	6B	7	Ahmed et al. 2021	1
SD	6D	2	Ahmed et al. 2021	0
SD	7A	10	Shahinnia et al. 2016, Liu et al. 2025, Ahmed et al. 2021	2
SD	7B	5	Ahmed et al. 2021	1
gs	2B	1	Wang et al. 2015	0
gs	2D	1	Wang et al. 2015	0
gs	4A	1	Wang et al. 2015	0
gs	6D	1	Wang et al. 2015	0
gs	7B	2	Wang et al. 2015	0
GCL	1A	1	Shahinnia et al. 2016	0
GCL	1B	5	Shahinnia et al. 2016, Liu et al. 2025	0
GCL	3B	1	Shahinnia et al. 2016	0
GCL	4B	8	Shahinnia et al. 2016, Liu et al. 2025	0
GCL	5B	3	Liu et al. 2025	0
GCL	7A	3	Shahinnia et al. 2016	1

GCL	7D	1	Shahinnia et al. 2016	0
GCW	2A	1	Liu et al. 2025	2
GCW	2B	1	Liu et al. 2025	0
GCW	5B	7	Liu et al. 2025	0
GCW	5D	1	Liu et al. 2025	2
GCW	7A	4	Liu et al. 2025	0

Table S8: The number of putative QTLs within a 10 Mbp region by chromosome and trait. A region was segmented by clustering marker position using complete-linkage in hierarchical clustering such that no marker in a region would have distance of more than 10Mbp. The 6 bolded rows indicate a region that may contain pleiotropic QTLs for stomatal traits.

Chromosome	Range of region (Mbp)	GCL	GCW	SA	SD	gsmax
1A	[7,14]	0	0	0	1	0
**1A**	**[372,380]**	**0**	**1**	**1**	**1**	**0**
1A	[515,520]	0	1	0	0	0
1A	[556,564]	0	1	0	0	0
1A	[573,579]	0	0	0	0	1
1B	[10,20]	0	1	0	0	0
1B	[412,417]	0	1	0	0	0
1B	[487,494]	0	0	0	0	1
1B	[577,586]	0	0	0	0	1
2A	[202,213]	0	0	0	1	0
2A	[360,371]	0	1	0	0	0
**2A**	**[429,434]**	**1**	**0**	**1**	**0**	**1**
2A	[447,455]	0	1	0	0	0
2A	[747,750]	0	0	0	0	1
2A	[762,769]	1	0	0	0	0
**2B**	**[153,161]**	**1**	**0**	**0**	**1**	**0**
2B	[431,438]	0	0	0	0	1
2B	[788,794]	0	0	0	0	1
2D	[63,72]	0	0	1	0	0
3A	[496,502]	1	0	0	0	0
3A	[543,549]	0	0	0	0	1
3A	[644,655]	0	0	1	0	0
3A	[713,721]	0	0	0	0	1
3A	[734,744]	0	0	0	1	0

4A	[116,122]	0	1	0	0	0
4A	[707,712]	1	0	0	0	0
**4B**	**[14,20]**	**0**	**1**	**0**	**1**	**0**
4B	[600,604]	0	1	0	0	0
5A	[585,593]	0	0	0	1	0
5B	[679,689]	0	0	0	0	1
**5D**	**[547,557]**	**0**	**2**	**1**	**1**	**0**
6A	[536,542]	1	0	0	0	0
6A	[569,578]	0	1	0	0	0
6A	[581,589]	0	0	0	0	1
6A	[619,623]	0	0	0	1	0
**6B**	**[21,32]**	**0**	**1**	**1**	**0**	**0**
6B	[121,131]	0	1	0	0	0
6B	[131,139]	2	0	0	0	0
6B	[462,472]	0	0	0	1	0
6B	[482,487]	0	1	0	0	0
6B	[726,731]	0	1	0	0	0
7A	[195,200]	0	0	0	1	0
7A	[345,354]	0	0	0	1	0
7A	[565,576]	1	0	0	0	0
7B	[108,114]	0	0	0	0	1
7B	[256,262]	2	0	0	0	0
7B	[556,564]	0	0	0	1	0
7B	[630,639]	0	1	0	0	0
7B	[738,749]	1	0	0	0	0
7D	[56,62]	0	0	1	0	0
